# Supplementary material for: Cohort profile: the open, prospective Community-Based chronic Care Lesotho (ComBaCaL) cohort – design, baseline chronic disease risk factors and hypertension and diabetes care cascades
Source: BMJ Open. 2025 Jul 25;15(7):e093852. doi: 10.1136/bmjopen-2024-093852 (PMC12306284; doi:10.1136/bmjopen-2024-093852)
Supplement: online supplemental file 2 [file bmjopen-15-7-s002.pdf]

Study Protocol

Community-Based chronic disease Care in rural Lesotho: The ComBaCaL cohort study

Type of Research Project	Research project involving collection of health-related data from persons

1 GENERAL INFORMATION

I. List of Project Leaders and other key persons involved in the study

The project leaders are qualified individuals by education and training and responsible for the whole project. All further key persons are also qualified by education and training to perform their assigned tasks and responsibilities.

II. Signatures

Study Title: "Community-Based chronic disease Care in rural Lesotho: The ComBaCaL cohort study"

The following project leaders have approved the protocol version 1.0, dated 22.09.2022, and confirm hereby to conduct the project according to the current version of the Declaration of Helsinki as well as all national legal requirements and guidelines as applicable.

Principal Investigators:

- I have read this protocol version 1.0, dated 22.09.2022, and agree that it contains all necessary details for carrying out this study. I will conduct the study as outlined herein and will complete the study within the time designated.
- I will ensure that all individuals and parties contributing to this study are qualified and I will implement procedures to ensure integrity of study tasks and data.
- I will provide copies of the protocol and all pertinent information to all individuals responsible to me who assist in the conduct of this study. I will discuss this material with them to ensure they are fully informed and trained regarding their activities within the study conduct.
- I will use only approved informed consent forms and will fulfil all responsibilities for submitting pertinent information to the Independent Ethics Committees responsible for this study.
- It is understood that this protocol will not be disclosed to others without prior written authorisation from the Project Leader or Sponsor, except where required by applicable local laws

Principal Investigators

National Principal Investigator

Statistician

Investigator

Table of contents

1 General Information 1
I. List of Project Leaders and other key persons involved in the study 1
II. Signatures 2
III. Abbreviations / Glossary of terms 5
IV. Synopsis 6
2 Background information 11
2.1 NCD burden 11
2.2 Decentralized chronic disease care 11
3 Objectives and purpose 13
3.1 Objectives 13
3.1.1 Specific objectives 13
3.2 Scientific justification of study population 13
4 Study design 15
4.1 Variables of interest 15
4.1.1 Cohort variables 15
4.1.2 Implementation outcomes 17
4.2 Measures to minimize bias 17
4.2.1 Blinding 17
4.2.2 Measurements 17
4.2.3 Randomization 17
4.3 Study duration and duration of participant's participation 17
4.4 Amendments 17
4.5 Withdrawal and discontinuation 18
4.5.1 Individual level 18
4.5.2 Study and village level 18
4.6 End of project 19
5 Selection of study participants 20
5.1 Selection of villages 20
5.2 Selection of CC-VHWs 20
5.3 Recruitment of participants 21
6 Study procedures 22
6.1 General Setting 22
6.2 Baseline 22
6.3 Follow-up 22
6.3.1 Regular follow-up 22
6.3.2 Clinical follow-up 22
6.4 Implementation assessments 23
7 Safety considerations 24
7.1 Definition and documentation of safety outcomes 24
7.2 Causality of SCE(SI)s and CESIs 25
7.3 Reporting of SCEs and CESIs 25
8 Statistics 26
8.1 Determination of sample size 26
8.2 General cohort statistics 26
8.3 Analysis of the ComBaCaL activities' effects on chronic disease care cascades 26
8.3.1 Hypothesis 26
8.3.2 Endpoints 26
8.3.3 Statistical methods 27
8.4 Handling of missing data 27
9 Description of data management 28
9.1 Specification of source documents 28
9.2 Data recording 28
9.3 Confidentiality and coding 28
9.4 Data security, access, archiving and back up 29
9.5 Data storage on public data repositories 29
10 Quality control and quality assurance 30
10.1 Supervision 30
10.1.1 Local supervision and data quality checks 30
10.1.2 Central data monitoring and quality checks 30
10.2 Translations - Reference language 30
10.3 Monitoring and auditing 30
10.4 Storage of biological material and related health data 31
11 Ethical considerations 32
11.1 Independent Ethics Committees (IECs) 32
11.2 Risk-benefit ratio 32
11.3 Participant information and consent 32
11.3.1 Household consent 32
11.3.2 Individual consent 33
11.3.3 Withdrawal on household or individual level 33
11.3.4 Service delivery for people declining consent 33
11.4 Participant confidentiality 33
11.5 Participants requiring particular protection 34

11.6 Participant compensation 34
11.7 Damage coverage / Insurance ..... **Error! Bookmark not defined.**
12 Funding 35
13 Dissemination of results and publication policy 35
13.1 Dissemination to scientific community 35
13.2 Information of community and policy makers 35
14 Appendix 36
14.1 Screening and diagnosis of aHT 36
14.2 Screening and diagnosis of (pre)DM 37
15 References 38

III. Abbreviations / Glossary of terms

aHT	Arterial hypertension
BMI	Body mass index
BG	Blood glucose
BP	Blood pressure
CC Nurse	Chronic Care Nurse
CC-VHW	Chronic Care Village Health Worker
CESI	Clinical event of special interest
cmRCT	Cohort multiple randomized controlled trials
ComBaCaL	Community-Based Chronic Disease Care Lesotho
CVDRF	Cardiovascular disease risk factor
DHMT	District Health Management Team
DKF	Department for Clinical Research
DM	Diabetes mellitus
EKNZ	Ethics Committee of Northern and Central Switzerland
FBG	Fasting blood glucose
HbA1c	Glycated haemoglobin
HIV	Human immunodeficiency virus
ICF	Informed Consent Form
IEC	Independent Ethics Committee
IT	Information technology
LMICs	Low- and middle-income countries
MoH	Ministry of Health
MTA	Material transport agreement
NCD	Non-communicable disease
NH-REC	National Health Research Ethics Council
RBG	Random blood glucose
SCE	Serious clinical event
TB	Tuberculosis
TwiC	Trial within cohort
UNAIDS	Joint United Nations Programme on HIV/AIDS
VHW	Village health worker
WHO	World Health Organization

IV. Synopsis

**Project Leaders**	Prof. Dr. Niklaus Labhardt<br>Alain Amstutz MD, PhD
**Study Title**	Community-Based chronic disease Care in rural Lesotho: The ComBaCaL cohort study
**Short Title/Study ID**	ComBaCaL cohort study
**Protocol Version and Date**	Version 1.0, 22.09.2022
**Study Category with Rationale**	Cohort study<br>Risk category A
**Background and Rationale**	Globally, non-communicable diseases (NCDs) are the leading cause of death and disability[1] with a particularly high burden in LMICs, where more than 75% of all premature NCD deaths occur.[2] In sub-Saharan Africa, the NCD burden has risen significantly over the past two decades, driven by the increasing prevalence of cardiovascular risk factors such as unhealthy diets, smoking, reduced physical activity, arterial hypertension (aHT), obesity, diabetes mellitus (DM), dyslipidemia, and air pollution.[3–6] It is anticipated that NCDs will overtake communicable, maternal, neonatal, and nutritional diseases combined as the leading cause of mortality in sub-Saharan Africa by 2030.[7]<br>Lesotho is a typical example of an African LMIC where NCDs are overtaking HIV/AIDS and other infectious diseases as major cause of disability, morbidity and early death[1].<br>Despite a drastic health worker shortage, particularly in the rural areas where the majority of the population lives, and the second-highest adult HIV prevalence globally (21.1%)[8] Lesotho has managed to reduce HIV transmission and AIDS-related deaths considerably.[9] This success is based on decentralized HIV testing and care, involving lay village health workers (VHWs) to deliver accessible and equitable services in urban and rural areas alike.[10,11] It has been demonstrated that decentralization and task shifting of healthcare services to lay healthcare workers have the potential to bring services closer to the community and to reduce access barriers such as transport costs, travel time and lacking awareness without compromising quality of care.[12–14] However, current community-based care delivery services in Lesotho and other sub-Saharan African countries are largely neglecting NCDs.[15] NCD care is located at health facilities where due to high workload, shortage of staff, lack of specific training and outdated practices, essential services often cannot be delivered adequately.<br>HIV and NCDs are chronic diseases and share several characteristics such as the asymptomatic initial phase, progression to complications with disability and early death, and need for life-long treatment. The Ministry of Health (MoH) of Lesotho has therefore proposed in its NCD strategic plan that lessons learnt from the HIV program should be taken up to similarly reduce the existing access barriers to NCD care.[16] However, two scoping reviews conducted by members of our group, have shown that the evidence on how and to what extent task shifting to lay workers can successfully be implemented for NCDs in sub-Sahara Africa is very limited.[17,18]

	In the open, prospective ComBaCaL cohort study we aim at generating evidence on community-based screening, diagnosis and management of uncomplicated aHT, DM and other chronic diseases by lay VHWs in a rural sub-Saharan African setting.
**Objectives**	• To establish an observational cohort (ComBaCaL cohort) with regular monitoring of chronic disease indicators and risk factors in Butha-Buthe and Mokhothlong districts in Lesotho that will be managed by lay Chronic Care Village Health Workers (CC-VHWs), supported by a dedicated tablet-based eHealth application<br>• To assess the prevalence of common chronic diseases and associated risk factors in the ComBaCaL cohort population and to monitor their development over time. Initial focus will be on aHT, DM, cardiovascular disease risk factors (CVDRFs) and HIV. Other conditions may be included at a later stage after submission of separate amendments<br>• To assess and describe implementation outcomes of the ComBaCaL cohort study, i.e. the feasibility, acceptability, appropriateness, resource use, costs, service coverage and quality of data collected and services provided<br>• To assess the effect of the ComBaCaL activities on condition-specific care cascade outcomes, such as screening coverage, disease awareness, linkage to care, engagement in care and disease control rates<br>• To prepare the ComBaCaL cohort as a platform for nested pragmatic trials (Trials within a Cohort, TwiCs) assessing chronic disease care interventions
**Cohort variables and implementation outcomes**	**Cohort variables**<br>The variables below will be collected at enrolment. Variables which may change over time will be reassessed during follow-up visits at intervals of around six months.<br>Household level:<br>• Number of household members<br>• Geolocation<br>• Socioeconomic indicators<br>Individual level adults (18 years and older):<br>• Sociodemographic characteristics, anthropometrics and brief medical history including HIV status<br>• Chronic disease indicators, such as blood pressure (BP), blood glucose (BG), HbA1c (if available), and self-reported drug intake for aHT or DM and based on those, presence of aHT and (pre)DM in the cohort population (see diagnostic criteria and algorithms in the appendix)<br>• Chronic disease risk factors, such as physical inactivity, unhealthy diet, alcohol, tobacco and other substance use<br>• Chronic disease care cascade indicators, such as screening coverage, disease awareness, linkage to and engagement in care, adherence to treatment and disease control levels for participants diagnosed with a chronic disease<br>• Occurrence of clinically relevant events (see section 7)

	Individual level adolescents (10 to 17 years):<br>• Date of birth, sex, anthropometrics, brief medical history<br>• Chronic disease risk factors, such as physical inactivity, unhealthy diet, alcohol, tobacco and other substance use<br>• Occurrence of clinically relevant events (see section 7)<br>Individual level children (9 years and younger):<br>• Date of birth, sex<br>• Occurrence of clinically relevant events (see section 7)<br>**Implementation outcomes**<br>• Acceptability, satisfaction and perceived appropriateness of ComBaCaL's community-based activities among participants, CC-VHWs and involved healthcare professionals<br>• Coverage outcomes, such as number of households visited per CC-VHW, number of individuals enrolled and assessed per CC-VHW, number of individuals newly diagnosed with a chronic condition, number of village inhabitants declining cohort consent and number of cohort participants refusing chronic disease screening or referral to health facility after diagnosis<br>• Quality outcomes of the CC-VHW activities, such as completeness of the data collected and adherence to clinical algorithms provided via the eHealth application<br>• Resource use and cost estimates of the CC-VHW activities
**Study Design**	ComBaCaL is designed as an open prospective cohort study enrolling inhabitants of randomly selected villages in the rural areas of Butha-Buthe and Mokhothlong districts in Lesotho. Each cohort village will be managed by a CC-VHW, who lives in the village and is being elected by the village population. CC-VHWs will be supported by a specifically developed eHealth application (ComBaCaL app) for data collection and clinical decision-making.<br>The ComBaCaL CC-VHWs in all villages will regularly assess chronic disease indicators and risk factors and offer linkage services if relevant conditions are detected.<br>For an implementation assessment of the cohort activities, metadata analysis of reports submitted via the ComBaCaL app and mixed-methods acceptability, satisfaction and perceived appropriateness assessments among different stakeholders will be used.<br>In addition to the cohort procedures described in this protocol, we plan to conduct community-based interventions within the ComBaCaL cohort villages in a cohort multiple randomized controlled trials (cmRCT) approach[19] also known as Trials within a cohort (TwiCs). Each TwiC will be submitted separately to the Independent Ethics Committees (IECs) with reference to this protocol.
**Inclusion/Exclusion Criteria**	Inclusion criteria village level:<br>• Village size of 40 to 100 households<br>• Village consent by village chief<br>• Possibility to recruit and train a CC-VHW from the village population who fulfils the eligibility criteria (outlined in chapter 5.2)<br>Inclusion criteria individual level:<br>• Primary residency in the respective village

	• Verbal household consent and written individual consent (or guardian consent for children and adolescents below the age of 18 years)
**Measurements and Procedures**	For the cohort recruitment, all households of selected villages will be contacted by the CC-VHWs in door-to-door visits. Consent will be sought first orally at household level from the household head or his/her representative in case of absence and then in written, electronic form at individual level.<br>At baseline, the variables of interest at household and individual level will be collected by the CC-VHWs. All adult participants will be screened for aHT via standardized screening algorithms using automated BP measurements (see appendix). Adult participants, 40 years or older or having a BMI equal or above 25kg/m^2 will be screened for (pre)DM according to standardized screening algorithms using capillary BG measurements (see appendix). HbA1c levels may be measured in participants diagnosed with (pre)DM (depending on access to and availability of HbA1c). Participants with clinical alarm signs or symptoms or any medical condition requiring further diagnostic work-up or treatment will be referred to the closest health facility or the respective district hospital.<br>Regular follow-up visits by the CC-VHWs among all cohort participants at intervals of six months will be conducted throughout the duration of the study. During the follow-up visits, the cohort census will be updated and the demographic and clinical data of existing participants will be reassessed. In addition to regular follow-up visits, visits based on clinical need will be conducted (e.g. to confirm the diagnosis after a first elevated screening result or for treatment monitoring after established diagnosis).<br>Clinically relevant events will be documented throughout the duration of the cohort follow-up by the CC-VHWs in dedicated electronic report forms within the ComBaCaL app (see section 7 for definitions).<br>To document the process of implementing and maintaining the cohort, we will conduct mixed-methods assessments about the acceptability, satisfaction and perceived appropriateness of the community-based activities among participants, CC-VHWs and involved healthcare professionals at different points in time. We will evaluate the quality of services provided and the data collected in the villages by analysing aggregated data and metadata of the reports submitted by the CC-VHWs via the ComBaCaL application.
**Number of Participants with Rationale**	In a first step, we aim to enrol inhabitants of around 100 (range 90-110) randomly selected villages in rural Lesotho. The estimated mean number of inhabitants per village is 200. All inhabitants will be approached for consent and all consenting individuals (assent plus guardian consent for adolescents (10-17 years), guardian consent for children <10 years) will be enrolled into the ComBaCaL cohort. Consent rate is expected to be high (ca. 80%). Thus, the number of cohort members is estimated to be around 16'000.<br>The number of participants was chosen based on available resources and with the aim to allow for meaningful TwiCs for the assessment of community-based chronic disease care delivery strategies.

**Study Duration**	The cohort will be followed-up for at least three years. Cohort follow-up might be prolonged if resources allow.
**Study Schedule**	First-Participant-In: January 2023<br>Last-Participant-Out: Earliest December 2025, later if resources for further maintenance of the cohort become available
**Study Centre(s)**	Scientific lead: Division of Clinical Epidemiology, University of Basel<br>Implementing partners: Ministry of Health Lesotho, SolidarMed Lesotho<br>Study sites: Pre-selected villages in rural areas of Butha-Buthe and Mokhothlong districts in Lesotho
**Statistical Analysis incl. Power Analysis**	As an open prospective cohort, ComBaCaL aims to provide observational data on chronic diseases and related risk factors at individual and household level in addition to operational indicators describing implementation aspects of the cohort.<br>A description of the cohort at different time points will be presented using descriptive statistics. Ordinal and binary variables will be presented as counts and proportions with 95% confidence intervals. Continuous variables will be described as medians and inter-quartile ranges and means with 95% confidence intervals.<br>We will conduct a pre-specified analysis to assess the effect of the ComBaCaL activities on chronic disease care cascades. We are hypothesizing that the ComBaCaL activities will have a positive effect on condition-specific care cascade outcomes, such as the screening coverage, disease awareness, linkage to care, engagement in care, adherence to treatment and disease control level) at six months (range 150-240 days) and twelve months (range 300-480 days) after enrolment.<br>In the future, we aim to use the ComBaCaL cohort as a platform for the implementation of pragmatic TwiCs. These TwiCs, including statistical analysis and sample size calculations will be detailed in separate protocols and submitted for ethics review with reference to this protocol.
**Ethical consideration**	This project will be carried out in accordance with this protocol and with principles enunciated in the current version of the Declaration of Helsinki[20], as well as all applicable national legal requirements and guidelines.<br>The protocol will be reviewed by the Ethikkommission Nordwest- und Zentralschweiz (EKNZ, Ethics Committee of Northern and Central Switzerland) and by the National Health Research Ethics Committee (NH-REC) of Lesotho.<br>Participation is voluntary, risks are minimal and consent can be withdrawn at any time. The evidence generated in this study aims at informing future national and international clinical guidelines and policies to improve chronic disease care in low-resource settings. Participants will benefit from improved chronic disease services in their villages through presence of trained eHealth supported and supervised CC-VHWs. Thus, the ComBaCaL study is likely to have a direct positive effect on health outcomes of participants as well as be generating evidence to improve context-specific chronic disease care on a longer perspective.

2 BACKGROUND INFORMATION

2.1 NCD burden

Globally, non-communicable diseases (NCDs) are the leading cause of death and disability with a particularly high burden in low- and middle-income countries (LMICs)[1] where more than 75% of all premature NCD deaths occur and where about 80% of deaths are caused by NCDs, resulting in a disproportionate health and socio-economic burden[21]. Arterial hypertension (aHT) is associated with 10.8 million annual deaths globally, mostly in LMICs' rural areas, where treatment rates are below 30%[3–6,22]. There is a lack of scientifically validated pragmatic and scalable prevention and care models in LMICs to make NCD screening and treatment equitably accessible[18]. In sub-Saharan Africa, the NCD burden has risen significantly over the past two decades, driven by the increasing prevalence of cardiovascular risk factors such as unhealthy diets, smoking, insufficient physical activity, aHT, obesity, diabetes mellitus (DM), dyslipidemia, and air pollution[3,4]. It is anticipated that NCDs will overtake communicable, maternal, neonatal, and nutritional diseases combined as the leading cause of mortality in sub-Saharan Africa by 2030[7].

Lesotho is a landlocked country within South Africa, a typical example of an African LMIC where NCDs are overtaking HIV and other infectious diseases (especially TB) as major cause of disability, morbidity and early death[7]. Of the adult population, 22% are affected by aHT and 6% by DM.[1,16]

2.2 Decentralized chronic disease care

Extensive evidence is showing that decentralization of healthcare services with task-shifting to community-based lay cadres may (cost)-effectively increase the access to care and improve health outcomes for a variety of diseases, especially in settings where professional healthcare workforce and financial health system resources are scarce.[12,23–26] Based on these findings, the World Health Organization (WHO) is promoting the strategic integration of community-based healthcare services in existing health systems[27] and the United Nations Programme on HIV/ AIDS (UNAIDS) has launched a plan to recruit 2 million community health workers in Africa to support such a strategy.[28]
Community-based lay health workers are bringing services closer to the community and are reducing access barriers such as transport costs, travel time and lacking awareness. In the spirit of "leave no one behind", community-based care offers more equitable and less stigmatized access to health services than facility-based care. In addition, it has the potential to strengthen civil society and create job opportunities in rural areas.[27]

As in many other sub-Saharan African countries, the health system in Lesotho is facing the challenges of lacking human and financial resources. As a countermeasure, the integration of lay healthcare workers into the existing health system structures has been adopted many years ago[15]. Despite a drastic health workers shortage in Lesotho (0.9 doctors and 10.2 nurses per 10,000 inhabitants, particularly in the rural areas where the majority of the population lives (77.6%)[29], and the second-highest adult HIV prevalence globally (21.1%)[8], Lesotho has managed to reduce HIV transmission and AIDS-related deaths considerably. This success is based on decentralized HIV testing and care, involving lower cadre healthcare workers and lay providers to deliver accessible and equitable services for the urban and rural population alike. The HIV programs in rural areas have demonstrated that in Lesotho, health care tasks can be successfully decentralized and shifted to lay village health workers (VHWs), who support and act as a link between the community and the clinics to decrease the burden on overwhelmed health facilities. Currently, the community-based health care delivery in Lesotho is focused on HIV and maternal and neonatal diseases, largely neglecting NCDs. Thus, NCD screening, diagnosis, management and prevention are located at the health facilities. However, due to high workload, staff shortages, lack of specific training and medication stock-outs, NCD services are often not delivered adequately at facility-level.

The Ministry of Health (MoH) of Lesotho has proposed in its NCD strategic plan that lessons learnt from the HIV program should be incorporated into the NCD care strategy and that delivery platforms should provide integrated HIV/NCD services.[30] Although various modelling studies from the region suggest that integrated service delivery can be cost-effective, robust evidence around community HIV/NCD delivery platforms and their key enablers is missing.[29, 30] To the best of our knowledge, no studies have been conducted or policy documents developed on how to provide pragmatic and scalable prevention and treatment models for NCDs in the context of a high communicable disease burden in Lesotho.

We plan to tackle Lesotho's chronic disease double burden, consisting of the longstanding HIV and the growing NCD epidemics, through a multi-disciplinary research and implementation partnership, the Community-Based chronic disease Care Lesotho (ComBaCaL) program. ComBaCaL aims at establishing and validating a community-based chronic disease care model involving ehealth-supported lay healthcare workers. The ComBaCaL cohort study provides the platform for the scientific assessment of the proposed community-based chronic disease care model.

3 OBJECTIVES AND PURPOSE

3.1 Objectives

The overall objective of the ComBaCaL cohort study and subsequent nested trials is to assess the impact of community-based, lay-led chronic disease screening and care interventions in rural Lesotho.
We aim to establish a prospective research and service delivery platform in rural Lesotho that is managed by eHealth-supported CC-VHWs providing regular chronic disease screening, monitoring and referral services. We want to assess implementation outcomes of the cohort as well as the effect of the cohort activities on disease-specific care cascades. Subsequently, we will develop nested trials to assess the effectiveness of specific chronic disease control interventions.

3.1.1 Specific objectives

- To establish a prospective, open cohort with regular monitoring of chronic disease indicators and risk factors in Butha-Buthe and Mokhothlong districts in Lesotho, that will be managed by lay CC-VHWs, supported by a dedicated eHealth application (ComBaCaL app)
- To assess the prevalence of common chronic diseases and associated risk factors in the ComBaCaL cohort population and to monitor their development over time. Initial focus will be on aHT, (pre)DM, CVDRFs and HIV, other conditions may be included at a later stage after submission of amendments to this protocol
- To assess and describe implementation outcomes of the ComBaCaL cohort study, i.e. the feasibility, acceptability, appropriateness, service coverage, resource use, costs and quality of the data collected and the services provided
- To assess the effect of the ComBaCaL activities, which include lay-led, eHealth supported, community-based screening, diagnosis and referral services for chronic diseases, such as aHT and (pre)DM on the condition-specific care cascade outcomes, such as screening coverage, disease awareness, linkage to care, engagement in care, adherence to treatment and disease control levels
- To prepare the ComBaCaL cohort as a platform for pragmatic TwiCs for the assessment of chronic disease control interventions

3.2 Scientific justification of study population

The ComBaCaL cohort study will be located in rural villages of Butha-Buthe and Mokhothlong districts in Lesotho. Lesotho is a typical example of an African LMIC where a developing health system is facing the heavy double-burden of the still highly prevalent infectious diseases HIV/AIDS and TB in combination with a rapidly spreading NCD epidemic[7].

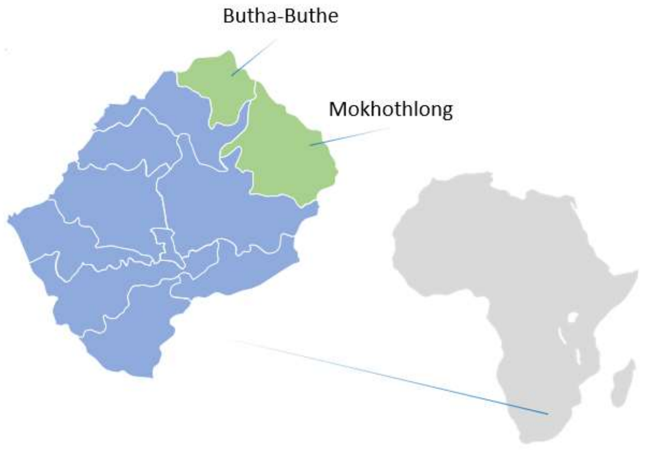

Figure 1 Map of Lesotho with the two districts Butha-Buthe and Mokhothlong

In the Lesotho health system, the VHW program plays a crucial role and has proven highly effective for the control of HIV/AIDS, especially for remote rural areas[15]. The Lesotho VHW program thus represents a meaningful starting point for implementation research on enhancing community-based healthcare intervention strategies and provides a setting that is representative for the health systems in many other LMICs, especially in sub-Saharan Africa, where lay worker-led care has a similar standing.
A recent systematic review assessing randomized controlled DM care trials in Africa has concluded that “there is a big lack of evidence in primary healthcare and rural settings, implementation research, pharmacological interventions, especially in poorer countries”.[33] Similarly, a scoping review conducted by some of the co-investigators of this protocol concluded that the overall evidence on community-based aHT care is low making it impossible to recommend or refute such models of care in sub-Sahara Africa[18]. This is the gap, the ComBaCaL cohort study is aiming to fill and that is justifying the choice for the rural setting in Lesotho where the need for local NCD implementation research has remained unaddressed so far.

4 STUDY DESIGN

The ComBaCaL cohort is an open prospective cohort enrolling inhabitants of randomly pre-selected villages in the rural areas of Butha-Buthe and Mokhothlong districts in Lesotho. At cohort initiation, we will enrol inhabitants of around 100 (range 90-110) villages. Each cohort village will be managed by a CC-VHW, who lives in the village and is being elected by the village population. CC-VHWs will be supported by the specifically developed tablet-based ComBaCaL app for data collection and clinical decision support for screening and diagnosis of chronic diseases, such as aHT and (pre)DM. CC-VHWs will be supervised by healthcare professionals of the respective catchment area's health facility and Chronic Care nurses (CC nurses).

The CC-VHWs in all villages will regularly update the cohort census, assess for indicators of chronic diseases, such as HIV, aHT, (pre)DM and associated risk factors and offer linkage and monitoring services for the respective conditions if required.

For an implementation assessment of the cohort activities, metadata analysis of reports submitted via the eHealth application, mixed-methods acceptability, satisfaction and perceived appropriateness assessments among different stakeholders will be used.

In addition to the cohort activities described in this protocol, we plan to assess the effect of community-based chronic disease care interventions within the ComBaCaL villages in a cohort multiple randomized controlled trials (cmRCT) approach[19,34,35] also known as trials within a cohort (TwiCs). These TwiCs are not part of this protocol, but will be submitted separately to the IECs with reference to this protocol.

4.1 Variables of interest

4.1.1 Cohort variables

All cohort variables will be assessed at baseline. Variables which may change over time will be reassessed during follow-up visits at intervals of around six months.

Household level:

- Number of household members. A household member is defined as recognized as such by the head of household or representative.
- Geolocation (GPS coordinates)
- Socioeconomic indicators (household wealth, food security and healthcare access)

Individual level, adults (≥18 years)

- Sociodemographic data:
  - Date of birth
  - Sex
  - Level of education
  - Income generating activity
- Chronic disease indicators (see appendix for detailed procedures):
  - Blood pressure (BP)
  - Blood glucose (BG) for those with BMI ≥25 kg/m^2 or age ≥40 years
  - HbA1c for those diagnosed with (pre)DM (depending on access to and availability of HbA1c)
  - Self-reported HIV status (or retrieved from Bukana (personal health booklet))
- Presence of aHT, (pre)DM (see diagnostic algorithms in the appendix) and HIV
- Chronic disease risk factors
  - Height, weight, BMI and abdominal circumference
  - Physical inactivity using the validated International Physical Activity Questionnaire Short Form (IPAQ-SF)[36], which has been adapted to the local context and language according to the IPAQ recommendations[37]

- Unhealthy diet using a shortened unquantified food frequency questionnaire adapted from an assessment tool for obesity used in South Africa[38]
- Alcohol, tobacco and other substance use using the Alcohol, Smoking and Substance Involvement Screening Test (ASSIST)[39] or a similar tool adapted to the setting
- Targeted medical history, including presence of chronic kidney disease, prior stroke, prior myocardial infarction (self-reported or retrieved from Bukana)

- Chronic disease care cascade indicators (will be assessed separately for each condition):
  - Screening coverage: number of participants who have been screened for aHT, (pre)DM and HIV during last three years (1095 days) among those who are screening-eligible (self-reported or retrieved from Bukana or ComBaCaL app, see appendix for screening eligibility criteria)
  - Disease awareness: number of participants with aHT, DM or HIV being aware of their condition (self-reported or retrieved from Bukana)
  - Linkage to care: number of participants with aHT, (pre)DM or HIV who had a check-up measurement (BG or HbA1C for (pre)DM, BP for aHT, VL for HIV[1]) or drug refill within the last 180 days for their condition (self-reported or retrieved from Bukana, ComBaCaL app, health facility register or VL database)
  - Engagement in care: number of participants with aHT, DM or HIV reporting drug intake and number of participants with aHT or (pre)DM actively following lifestyle recommendations (self-reported)
  - Adherence level of participants being engaged in care for aHT, DM or HIV
  - Disease control level: number of participants with aHT, DM or HIV who are reaching disease-specific treatment targets (BP <140/90 mmHg for aHT, FBG < 7mmol/l and/or HbA1C < 7.0% for DM, documented viral suppression for HIV in the last 12 months)
- Occurrence of clinically relevant events (see section 7.1)

Individual level, adolescents (10 - 17 years)

- Sociodemographic data:
  - Date of birth
  - Sex
  - Level of education
- Chronic disease risk factors
  - Height, weight & BMI
  - Physical inactivity using the validated International Physical Activity Questionnaire Short Form (IPAQ-SF)[36] which has been adapted to the local context and language according to the IPAQ recommendations[37]
  - Unhealthy diet using a shortened unquantified food frequency questionnaire adapted from an assessment tool for obesity used in South Africa[38]
  - Alcohol, tobacco and other substance use using an age-adapted version of the ASSIST questionnaire[40–43] or a similar tool
- Occurrence of clinically relevant events (see section 7.1)

Individual level, children (< 10 years)

- Sociodemographic data:
  - Date of birth
  - Sex
- Occurrence of clinically relevant events (see section 7.1)

[1] For HIV viral load, the interval is 12-monthly as per National Guidelines of Lesotho

4.1.2 Implementation outcomes

For an evaluation of the ComBaCaL cohort implementation, the following indicators will be assessed:

- Feasibility, acceptability, satisfaction and perceived appropriateness of ComBaCaL's community-based chronic disease care activities among participants, CC-VHWs and involved healthcare professionals using mixed-methods assessments
- Coverage indicators, such as number of households visited by CC-VHW, number of individuals monitored by one CC-VHW, number of individuals newly diagnosed with a chronic condition, number of villages inhabitants refusing community-based chronic disease screening or referral to health facility for further management after diagnosis
- Resource use and cost of the ComBaCaL activities, including time-and-motion studies among CC-VHWs
- Quality indicators of the community-based activities, such as completeness of the data collected by CC-VHWs and adherence to clinical algorithms provided via the eHealth application

4.2 Measures to minimize bias

4.2.1 Blinding

No blinding is foreseen for data collection in the cohort and implementation study. Clinically relevant events will be classified based on clinical data collected by the CC-VHW and the supervising CC nurse by a physician blinded to the name of the village, CC-VHW or CC nurse (see section 7.1).

4.2.2 Measurements

Processes for measurements of clinical outcomes and for the diagnosis of targeted diseases, such as aHT and (pre)DM will be standardized and closely supervised. The screening and diagnostic algorithms for aHT and DM (see appendix) will be coded into the ComBaCaL app ensuring that the same measurement procedures and diagnostic criteria will be applied for all participants. All CC-VHWs will undergo a baseline as well as regular refresher trainings with emphasis on correct BP and BG measurements as well as interviewing techniques and data entry into the ComBaCaL app. Further, they receive individual supervision and mentoring feed-back by the CC nurse. Health center nurses and CC nurses will undergo a training adapted to their supervisory tasks. For the duration of the study there will be regular field visits by supervising CC nurses to ensure that procedures for data collection are correctly followed by all involved CC-VHWs.

4.2.3 Randomization

No randomization is planned as part of the cohort procedures described in this protocol.
However, the selection of the ComBaCaL villages among all rural settlements in Butha-Buthe and Mokhothlong is done randomly to avoid selection bias. See section 5.1 for details.

4.3 Study duration and duration of participant's participation

The cohort will be followed-up for at least three years (36 months). The cohort follow-up may be prolonged beyond the three-year horizon if resources would become available. We plan to enrol first participants in January 2023.

4.4 Amendments

Substantial changes to the project set-up, the protocol and relevant project documents will be submitted to NH-REC and EKNZ for approval before implementation while minor amendments will be submitted to NH-REC only. Protocols of TwiCs nested within the ComBaCaL cohort population will be submitted to both involved IECs with reference to this protocol.

4.5 Withdrawal and discontinuation

4.5.1 Individual level

Participants can withdraw cohort consent at any time without being asked justification. The possibility to re-enter the cohort later through contacting the local CC-VHW will be offered. Data collected until the time of withdrawal will be included in the analysis.
The study team will not discontinue individual participants.
Importantly, participants may refuse specific parts of the assessments/screenings at enrolment or follow-up but remain in the cohort study. For example, a participant may refuse blood glucose measurement but agree to the other assessments.

4.5.2 Study and village level

The village chief may request withdrawal of a village via the local CC-VHW. The study team will then get in contact with the village chief to inquire the reasons for the intended withdrawal. If needed, a community gathering ("Pitso") will be held. If after discussion between the village chief, the community and the study team, the request for withdrawal persists, the data collection for the ComBaCaL study will be stopped in the village while the CC-VHW will continue with the routine activities as part of his/her task assigned by the MoH.
After withdrawal of village consent in one or more villages, the study team may replace withdrawing villages by other villages randomly selected from the list of rural villages not yet included in any other study in Mokhothlong and Butha-Buthe districts (see section 5.1).
If in a village, the CC-VHW is not able or willing to continue his/her tasks for the ComBaCaL study (i.e. due to death, migration, personal reasons, rejection by village chief or village population), the CC-VHW will be replaced while the village will remain in the study.
The Principal Investigators in consultation with Co-Investigators may choose to pause or discontinue the entire study or certain villages.

The reasons to pause or discontinue the entire study include the following:

- Insufficient funding to continue the study
- Significant opposition by local health authorities
- Safety or other ethical concerns
- Alteration in accepted clinical practice, national policy or scientific evidence that make the continuation of the study unwise
- Insurmountable technical or organizational problems

The reasons to pause or discontinue individual villages include the following:

- Insufficient funding to continue the study in all villages
- Significant opposition by local health authorities
- Safety or other ethical concerns
- Insurmountable organizational problems
- Impossibility to recruit a CC-VHW from the village population in case replacement of the initially recruited CC-VHW is required

The Principal Investigators would provide the project partners and the Co-Investigators written notice submitted at a reasonable time in advance of the intended discontinuation or pause. If the Principal Investigators choose to terminate or pause the study for safety reasons, they will immediately notify all investigators and subsequently provide written instructions for study termination. Co-investigators may pause the study in certain villages in case of safety concerns without written notice in advance. If Co-Investigators wish to pause or discontinue the study or certain villages, they may address the request to pause or discontinue in written form to the Principal Investigators. Co-Investigators may not pause or discontinue the study or certain villages for other reasons than safety concerns without consulting the Principal Investigators.

4.6 End of project

At the closure of the study or premature termination, all study data will be locked and archived. The electronic database will be locked and a complete study dataset will be transferred to the statistician and the Principal Investigators through a secure channel. The study data will be stored by the Department of Clinical Research (DKF) at the University Hospital Basel on a secure server for a minimum of 10 years and be destroyed thereafter (see section 9.4).

5 SELECTION OF STUDY PARTICIPANTS

5.1 Selection of villages

Participants of the ComBaCaL cohort will be recruited in randomly selected rural villages in Mokhothlong and Butha-Buthe districts in Lesotho. Based on the 2016 Lesotho Population and Housing Census[44], a total of 675 rural village-clusters have been identified in Mokhothlong (321) and Butha-Buthe (354) districts. Villages that were too small and remotely isolated, or were government settlements (e.g. informal mining village or other construction project) or belong to the area that will be flooded due to the construction of the Polihali dam were excluded. Out of the 675 rural villages, 70 (35 per district) were excluded because they were involved in other chronic disease related studies. In particular, 60 (30 in each district) were part of a population-based NCD prevalence survey and burden assessment (NH-REC ID 139-2021) and 10 (5 in each district) are part of the ComBaCaL pilot study (NH-REC ID 176-2021). Out of the 605 (319 in Butha-Buthe, 286 in Mokhothlong) remaining villages, 110 (55 per district) were randomly sampled, stratified by district and access to health facility (easy versus difficult access, defined as needing to cross a mountain or river or travel >10 km to the nearest health facility), by a statistician not involved in the study. The 110 villages will be assessed for eligibility applying the following criteria:

Inclusion criteria village level:

- Village size of 40 to 100 households
- Village consent obtained from village chief
- Possibility to identify or recruit a CC-VHW from the village population meeting the following requirements which are largely in line with the criteria of the Lesotho VHW Program Policy:
    - Criteria of the Lesotho Village Health Program Policy[45]:
        - Having primary residence in the village (according to village chief)
        - Having a proven record of trustworthiness in the resident village
        - Having proven ability to maintain confidentiality on public matters
        - Being aged between 20 and 50 years
        - Being able to provide written reports and being able to do basic mathematical calculations
        - Having at least educational level equivalent to high school leaving certificate (Junior Certificate)

    - Additional ComBaCaL criteria:
        - Having the ability and willingness to work with a tablet-based eHealth tool
        - Having good social and communication skills
        - Having the ability and willingness to interact with health professionals and the village population
        - Being able to speak, understand and write in English
        - Having successfully completed the ComBaCaL CC-VHW training including final assessment

5.2 Selection of CC-VHWs

Prior to cohort recruitment, in each ComBaCaL cohort village, one CC-VHW will be appointed. In villages where an existing VHW has the qualifications and capacity to fulfil the tasks associated with the ComBaCaL activities, he/she will be approached for collaboration in the project. In villages, where there is no VHW or where the existing VHW does not have the qualifications or capacity to fulfil the ComBaCaL tasks or where the existing VHW is declining the offer of collaboration, a new VHW will be selected through a participatory process involving the study team, the MoH district health management team (DHMT) and the village population: The study team together with the local DHMT will send a letter explaining the CC-VHW criteria as outlined above to the village committee composed of the chief, the area councillor and existing VHWs if applicable. The village committee will select three candidates among

the village inhabitants meeting the criteria. Among these three candidates, one will be elected to become the CC-VHW by the village population during a community gathering ("pitso").
The selected CC-VHWs will undergo a targeted training covering basic pathophysiological concepts, screening, diagnosis and basic life-style counselling for chronic diseases, such as aHT and DM, as well as technical trainings on the devices and tools used, especially on correct BP and BG measurements and data entry into the tablet-based ComBaCaL eHealth application.
Additionally, CC-VHWs may undergo the standard six-week VHW training as per the Lesotho Village Health Program Policy.[45] ComBaCaL specific activities may be started independent of whether the standard VHW training has been completed or not.

5.3 Recruitment of participants

All households in selected villages will be visited by the local CC-VHW in a door-to-door cohort recruitment campaign. All present household members will be asked for consent to participation in the ComBaCaL cohort study. Details regarding the two-stepped consent procedure are outlined in section 11.3. The number of absent household members will be registered and the VHW will return to all households with absent members within the following days to complete recruitment.
The ComBaCaL cohort is an open cohort. Thus, at any follow-up visit, new inhabitants of the selected villages will be approached for consent and be enrolled into the cohort if consenting while inhabitants moving out of the village or deceased inhabitants will be removed from the active cohort population.

Inclusion criteria individual level:

- Having primary residence in the village (self-reported at time of enrolment)
- Being able and willing to consent to participation or in case of individuals aged below 18 years, a caregiver who provides consent (with participant's assent for adolescents)

6 STUDY PROCEDURES

6.1 General Setting

Measurements and data entry will be conducted by CC-VHWs. The CC-VHWs will be equipped with the essential tools required for chronic disease monitoring in the community (i.e. BP machines, scales, measuring band, glucometers, and urine dipsticks). They will undergo a theoretical and practical training covering all aspects required for correct data collection and chronic disease screening, diagnosing, referral and counselling services. At every visit, the CC-VHW will screen participants for warning signs and symptoms (i.e. shortness of breath, severe headache, chest pain, new-onset confusion, impaired consciousness, severely impaired general state of health) and refer participants to the closest health centre in case of any danger-sign. The CC-VHWs will be continuously monitored and supervised by health centre nurses of the respective village's catchment area, mainly through direct interaction during monthly VHW meetings and by CC nurses through field visits, remote interaction via phone calls or messages sent via the ComBaCaL app and through direct contact during the monthly VHW meetings at the health centre.

The CC-VHWs are embedded within the Lesotho MoH VHW program and may during the project period be trained and equipped to provide further routine services in their communities. The supervision of these routine tasks outside the ComBaCaL activities will be provided within the existing VHW framework by health centre nurses and will not be influenced by the ComBaCaL activities.

6.2 Baseline

After recruitment, all variables of interest (see section 0) at household and individual person level will be collected.

All adult participants will be screened for aHT applying a standardized, evidence-based screening algorithm using automated BP measurements (see appendix). Participants aged 40 years or more or participants aged 18 years and more and having a BMI of equal or above 25kg/m^2, will be screened for (pre)DM applying a standardized, evidence-based screening algorithm using capillary BG measurements (see appendix). Participants, in which a relevant condition is detected will be referred to the closest health facility or the district hospital for further work-up and treatment.

6.3 Follow-up

6.3.1 Regular follow-up

Follow-up visits at intervals of around six months will be conducted. During the regular follow-up visits, the CC-VHWs will update the variables of interest collected at baseline. In addition to the updates of individual participant's data, the cohort census will be updated through registration of new households or new members of previously registered households as well as removal of emigrated or deceased participants from the active cohort population. During the regular follow-up, CC-VHWs will inquire about the occurrence of clinically relevant events (see section 7.1) since the previous visit (including screening of the personal health booklet, Bukana, for documentation of respective events). All clinically relevant events will be documented in a dedicated electronic case report form within the ComBaCaL app. All reports of clinically relevant events will be reviewed and validated by the supervising CC nurses and forwarded to the study physician.

6.3.2 Clinical follow-up

In addition to the regular follow-up visits, the CC-VHWs will conduct follow-up visits according to the clinical algorithms provided in the appendix. Clinical follow-up visits entail confirmatory BP and BG measurements for the diagnosis/exclusion of DM and aHT in case of elevated screening values detected at baseline or during regular follow-ups as well as monitoring of participants with established diagnosis of aHT or DM.

Furthermore, CC-VHWs will document census updates and clinically relevant events any time during the cohort follow-up when being informed thereof independent of scheduled visits.

6.4 Implementation assessments

For the assessment of satisfaction, acceptability and perceived appropriateness of the community-based activities among different stakeholders, questionnaires will be presented via the ComBaCaL application. The questionnaires may be self-reporting (for CC-VHWs and involved healthcare professionals) or will be administered by CC-VHWs or other study staff (i.e. for participants, CC-VHWs or involved healthcare professionals). Additionally, qualitative research approaches, such as focus group discussion or semi-structured interviews may be applied to gather information among participants, CC-VHWs or involved healthcare professionals.

7 SAFETY CONSIDERATIONS

7.1 Definition and documentation of safety outcomes

Clinical event of special interest (CESI)	Clinical event that is consistent with chronic disease complications, such as<br>• Myocardial infarction<br>• Stroke<br>• Symptomatic heart failure<br>• Chronic kidney disease<br>• Diabetic foot syndrome<br>• Peripheral arterial disease<br>• Hypertensive urgency or emergency<br>• Hyperglycemic emergency<br>• Visual impairment or blindness<br>• Peripheral neuropathy<br>• HIV/AIDS related event<br><br>Clinically relevant event possibly related to chronic disease treatment, such as<br>• Intolerance reaction against chronic disease medication leading to discontinuation of the medication concerned (including allergic reactions, drug interactions or rare severe side effects such as lactic acidosis)
Serious clinical event (SCE)	Clinical event that:<br>• Results in death<br>• Requires hospitalization<br>o Hospitalizations due to uncomplicated delivery are not considered as SCE<br>• Results in persistent or significant disability or incapacity<br>• Consists of a congenital anomaly or birth defect
Serious clinical event of special interest (SCESI)	SCE consistent with chronic disease complications, such as<br>• Myocardial infarction<br>• Stroke<br>• Symptomatic heart failure<br>• Chronic kidney disease<br>• Diabetic foot syndrome<br>• Peripheral arterial disease<br>• Hypertensive urgency or emergency<br>• Hyperglycemic emergency<br>• Visual impairment or blindness<br>• Peripheral neuropathy<br>• HIV/AIDS related event<br><br>SCE possibly related to chronic disease treatment, such as<br>• Intolerance reaction against chronic disease medication leading to discontinuation of the medication concerned (including allergic reactions, drug interactions or rare severe side effects such as lactic acidosis)

CC-VHWs will be trained to screen for and to recognize SCE(SI)s and CESIs and to document them in a dedicated form in the ComBaCaL app.

CC-VHWs may solicit SCE(SI)s and possible CESIs in the following ways:

- Reporting by participants or friends or relatives after inquiry by CC-VHWs during scheduled CC-VHW visits
- Active reporting by participants or friends or relatives outside of scheduled CC-VHW visits (scheduled visits are defined as visits being triggered through the ComBaCaL app based on regular or clinical follow-up algorithms or visits being assigned by supervisors)
- Clinical observation of CC-VHW during or outside scheduled CC-VHW visit
- Screening of participants' personal health booklets (Bukanas)
- Reporting by staff from health care facilities or other VHWs

Participants, friends, relatives and health centre nurses will be encouraged to notify the CC-VHW about relevant medical events during the cohort follow-up. Many CESIs (i.e. chronic kidney diseases) cannot be recognized clinically without access to further diagnostic tools (i.e. laboratory). For these cases, the CC-VHW will primarily document the results of investigations conducted and diagnosis made by healthcare professionals from entries in the Bukana or reports communicated by health centre nurses during the VHW meetings. If needed, the CC nurse may inquire further details at the facilities where the participant was treated for the SCE(SI). On the other hand, the CC-VHW may through her/his continuous presence in the village capture events (i.e. death after stroke or myocardial infarction) that would otherwise remain undocumented.

No other events than SCE(SI)s and CESIs will be documented and reported. All SCE(SI)/CESI reports that are submitted by CC-VHWs will be reviewed, validated and if required completed and/or corrected by the supervising CC nurse in the ComBaCaL app within 30 days after submission of the report. Deaths for which the cause cannot be identified by the CC nurse based on the initial report, he/she will follow up with a verbal autopsy using the Population Health Metrics Research Consortium's Shortened Verbal Autopsy Instrument.[46]

The anonymized SCE(SI)/CESI reports will subsequently be submitted on a monthly basis to the study physician who will remain blinded to the village, CC-VHW or CC nurse related to the report submission. The study physician will classify the reported cases as SCE other than SCESI, SCESI, CESI or none of the three if sufficient clinical information for classification is available. If the clinical information available is not sufficient for classification of the case, the study physician will request the study Data Manager to unblind the respective report and then contact the responsible CC nurse and/or CC-VHW and ask for collection of further data.

7.2 Causality of SCE(SI)s and CESIs

The ComBaCaL cohort study does not entail any intervention other than screening, diagnosing, counselling and referral of participants with relevant conditions. Therefore, a direct causation between the study activities and an SCE(SI) or CESI is highly unlikely. An indirect causation (for example a car accident on the way to the health centre or a drug reaction after prescription of chronic disease treatment after referral) is possible. No causality assessment will be performed for reported SCE(SI)s and CESIs.

7.3 Reporting of SCEs and CESIs

A list of all SCE(SI)s and CESIs will be submitted to the Principal Investigators on a quarterly basis and to the NH-REC along with the yearly reports.

8 STATISTICS

8.1 Determination of sample size

This is an open prospective cohort study and no sample size calculation is performed. In a first step, we aim to enrol inhabitants of around 100 villages. Assuming an average of 200 eligible inhabitants per village and a high participation around 80%, we estimate that we will reach a cohort population of around 16’000 participants. We have secured the funding to pursue a cohort of this size for at least three years.
Sample size calculation for future projects nested in the cohort (e.g. TwiCs) will be detailed within the corresponding sub-protocols.

8.2 General cohort statistics

The variables collected within the ComBaCaL cohort will be summarized using descriptive statistics such as counts, proportions with 95% confidence intervals, medians and interquartile ranges and means with 95% confidence intervals at time point of collection as defined. To establish the care cascade, proportions will be derived by dividing the number of occurrence of events at a specific stages of the cascade by the population at risk at the corresponding stage.

Statistical methods for future projects nested in the cohort will be part of the corresponding sub-protocols and separate statistical analysis plans.

8.3 Analysis of the ComBaCaL activities’ effects on chronic disease care cascades

The ComBaCaL activities with chronic disease screening and referral services are likely to have an effect on care cascade outcomes compared to the current standard of care without active screening at community-level. We will conduct a pre-specified analysis to assess the effect of the ComBaCaL on chronic disease care cascades.

8.3.1 Hypothesis

Care cascade outcomes for aHT and (pre)DM will improve after introduction of the ComBaCaL activities in the respective villages compared to baseline.

8.3.2 Endpoints

- Screening coverage for aHT and (pre)DM six months (150-240 days) and twelve months (300-480 days) after enrolment, defined as the proportion of screening-eligible participants that have been screened for the respective disease within the previous 3 years (1095 days, self-reported or retrieved from Bukana or ComBaCaL app)
    - Screening eligibility for aHT: Aged 18 years and above
    - Screening eligibility for (pre)DM: Aged 40 years and above or BMI of 25 kg/m^2 or above (adults only)
- Disease awareness for aHT and (pre)DM six months (150-240 days) and twelve months (300-480 days) after enrolment, defined as the proportion of participants diagnosed with aHT or (pre)DM that are aware of their condition (self-reported and/or retrieved from Bukana)
- Linkage to care for aHT and DM six months (150-240 days) and twelve months (300-480 days) after enrolment, defined as the number of participants with aHT or (pre)DM who (re)started drug treatment for their respective condition since enrolment
- Engagement in care for aHT and DM six months (150-240 days) and twelve months (300-480 days) after enrolment, defined as the number of participants with aHT or (pre)DM who had a check-up measurement (BG or HbA1C for (pre)DM, BP for aHT) or drug refill within the last 180 days for their condition (self-reported or retrieved from Bukana, ComBaCaL app or health facility register)

- Level of adherence to drug treatment for aHT and DM six months (150-240 days) and twelve months (300-480 days) after enrolment
- Disease control level for aHT and DM six months (150-240 days) and twelve months (300-480 days) after enrolment, defined as the number of participants with aHT or DM who are reaching disease-specific treatment targets (BP <140/90 mmHg for aHT, FBG < 7mmol/l and/or HbA1C < 7.0% for DM)

8.3.3 Statistical methods

We will test for difference in proportion of the different element of the cascade between the different time points (enrolment, six months and twelve months) using chi-square tests.

8.4 Handling of missing data

Missing data might be populated by additional information coming from relevant external registries. Multiple imputations techniques might be used to impute missing adjusting factors in subsequent analyses and will be detailed, if applicable, in the corresponding sub-protocols.

9 DESCRIPTION OF DATA MANAGEMENT

9.1 Specification of source documents

Data collected in the villages will be entered directly into the tablet-based ComBaCaL application by the local CC-VHW. Thus, the electronic forms within the ComBaCaL application serve as source documents in most cases. If required, the CC-VHW may extract clinical data from the patient's personal health booklet (Bukana) or from clinical reports and registers at the health facilities. For participants living with HIV the personal ART number will be collected to retrieve VL results from the viral load data base (NH-REC ID 134 2016). These sources may be important for documentation if participants are not able to provide the exact clinical information orally. The Bukana will always remain with the patient and paper-based or electronic routine clinical reports and registers will remain at the health centres.

9.2 Data recording

All data collected in the villages will be directly entered using the tablet-based ComBaCaL application with regular synchronization to a secure server (hosted at University Hospital of Basel) by trained CC-VHWs.

The ComBaCaL app is based on the open source Community Health Toolkit (CHT) Core Framework, the "most widely-used open source software toolkit designed specifically for community health systems".[47] Within the CHT Core Framework, a user hierarchy reflecting relevant healthcare cadres and facilities can be configured as well as a variety of tools such as task scheduling, clinical decision support and messaging services. According to the configured user hierarchy, access levels are defined: a CC-VHW will have access to the data collected in his/her village, a health centre nurse will have access to the data of the villages in her/his catchment area and a CC nurse will have access to the data collected by the CC-VHWs she/he is supervising. Adapted to the realities of community-based healthcare delivery, CHT defines a household level between village and individual participants. All individual participants are allocated to a household for which a household head is defined.

Follow-up visits will be prompted as tasks with a due date defined within the ComBaCaL app based on the algorithms encoded. This feature will minimize number of missing visits and enable efficient work planning for CC-VHWs and uncomplicated supervision of pending and upcoming tasks.

Besides the task scheduling, efficient remote supervision is facilitated within CHT through a report validation function and messaging services.

9.3 Confidentiality and coding

The data collected by CC-VHWs and documented in the ComBaCaL app will be used for clinical decision-making, central supervision, programme monitoring and research at the same time. The data collected will be handled with uttermost discretion and only be accessible to authorized personnel who require the data to fulfil their clinical or research duties. Each CC-VHW will have a single, password-protected tablet. Due to the use of the data collected for clinical decision support, contact and clinical data will both be collected in the ComBaCaL app. The app contains a specific contact form capturing personally identifying information such as name, date of birth and contact details for the purpose of identification by CC-VHWs and nurses involved in clinical care. Identifiable data will only be accessible by the respective CC-VHW, the supervising health centre and CC nurse and the study Data Manager overseeing the application's database, and it will not be included in any exported clinical data or metadata. Study participants are automatically allocated a unique participant number, which will be used to identify participants in all data exports containing clinical information. The investigators will respect participants' privacy according to all applicable privacy laws. Only anonymized study data will be published in scientific journals and presented at scientific meetings and conferences. All participants' personal and medical information are confidential and disclosure to third parties is prohibited. Access to identifiable data for the purpose of data quality control may be granted upon request to the Principal Investigators and other members of the study team responsible for data quality control.

9.4 Data security, access, archiving and back up

The study data will be stored on secure servers during the duration of the study. After termination of the study, the database will be locked and the data will remain on secure severs at the University Hospital Basel, where they will be stored for a minimum of 10 years and be destroyed thereafter.

9.5 Data storage on public data repositories

Following the FAIR principles[48], anonymized datasets of cohort analyses will be deposited on an openly accessible data repository, such as zenodo.org[49].

10 QUALITY CONTROL AND QUALITY ASSURANCE

10.1 Supervision

10.1.1 Local supervision and data quality checks

Data will be collected by the CC-VHWs and documented directly in the tablet-based ComBaCaL app. Direct supervision of CC-VHWs and data quality checks will be ensured by two complementary cadres. In the first cadre, routine health centre nurses will provide clinical supervision for the CC-VHWs in their catchment area through a supervisor login of the ComBaCaL app, which will allow them to review data collected and to send messages for queries to individual CC-VHWs. Furthermore, health centre nurses will provide mentoring, training and supervision in monthly meetings at the health centre where CC-VHWs will participate together with other VHWs.

In the second cadre, CC nurses will complement the supervision provided by routine health centre nurses. CC nurses are registered nurses who will be trained specifically on study procedures and on the management of DM, aHT and other chronic diseases. Each CC nurse will be responsible for the supervision of around 25 CC-VHWs. The CC nurses will regularly review and authorize the reports uploaded by the CC-VHWs in the ComBaCaL app. Additionally, they will conduct regular field visits in the villages for direct supervision and mentoring of the CC-VHWs and they will also be regularly present at the monthly VHW meetings at the health centres where they will provide NCD-specific training and mentoring for health centre nurses and CC-VHWs.

10.1.2 Central data monitoring and quality checks

In addition to the local supervision and data quality checks, regular data quality checks will be run by the study Data Manager on the overall database and anonymized, aggregated data will be shared with the study team for discussion of data quality issues. Queries will be communicated to the respective CC-VHWs either via messages sent within the ComBaCaL app or via the CC nurses.

10.2 Translations - Reference language

The reference language of the study documents is English. Official languages in Lesotho are English and Sesotho. No Sesotho translation will be provided for the study protocol and the electronic forms in the ComBaCaL app, as all staff of responsible IECs and the study team including the CC-VHWs are literate in English. The electronic patient information and ICFs as well as the paper-based patient information leaflets will be translated from an English master document into Sesotho and provided in both languages. The quality of translation will be ensured by back-translation as well as by quality and comprehension checks by bilingual individuals not involved in the translation.

10.3 Monitoring and auditing

Members of the Ministry of Health or the NH-REC may visit the research sites. To ensure appropriate organization of the visits, they may inform the principal investigators at least 14 days before the planned visit. Direct access to the source data and all project related files and documents will be granted on such occasions if feasible (access to participant-based Bukana might not always be possible).

Principal investigators and co-investigators may conduct site-visits, i.e. visiting certain villages to monitor adherence to the study protocol, verify correct conduction of study procedures or clarify uncertainties regarding data quality.

The data management team will conduct regular central monitoring at least on quarterly basis, monitoring data accuracy and completeness, progress in enrolment, adherence to clinical algorithms, occurrence of CESIs, and follow-up visits.

Systematic site visits by external monitors are not foreseen as visiting all 100 villages by external monitors would be resource intensive and because given that all key data are directly collected in the ComBaCaL app source-verification can be done on distance through the central monitoring. However, in case central monitoring should raise concerns or red flags regarding protocol adherence or data quality in specific villages, the investigators may organize external monitoring site visits of these villages.

10.4 Storage of biological material and related health data

Plasma or dried blood spot samples of cohort participants may be collected for storage and future analysis. Samples collected fall under the biobank agreement ("Biobanking regulations, v2.0") approved by the ethics committees in Lesotho and Switzerland. Samples will be stored in biobanks in Butha-Buthe or Mokhothlong districts in Lesotho. Samples may only be exported for analysis after submission and approval of a material transport agreement (MTA) by the NH-REC. No human genome analysis will be conducted on the samples collected. Results of analysis that are relevant for the health of participants will be communicated to participants. Samples will be destroyed latest five years after termination of the study.

11 ETHICAL CONSIDERATIONS

11.1 Independent Ethics Committees (IECs)

This protocol and any protocol amendments, the informed consent form (ICF), and all other forms of participant information related to the study and any other necessary documents will be reviewed and approved by the National Health Research Ethics Council (NH-REC) of Lesotho and by the Ethics Committee of Northern and Central Switzerland (EKNZ) before implementation.

11.2 Risk-benefit ratio

There is no substantial health risk associated with participation in the ComBaCaL cohort. The chronic disease screening and diagnosis offered will be conducted in line with international recommendations. All participants found to be at risk for a relevant medical condition will be referred to local health facilities for professional work-up and care as per national standard of care.
Data collection will entail questionnaires, automated BP measurements, capillary BG and HbA1C measurements, and urine dip stick analysis if clinically indicated. None of the study components has the potential to cause significant harm to participants. The capillary blood collection might cause slight discomfort but no serious complications.
No personalized data of participants will be shared with people other than directly involved study team members if not agreed upon by the participant.
The access to guideline-conform active community-based chronic disease screening with linkage services will likely increase early case detection and thus improve access to potentially life-saving treatment. Additionally, regular follow-up with monitoring in the community by CC-VHWs is likely to improve chronic disease care for participants compared to standard clinic-based care.
The evidence generated in this study may inform future national and international clinical guidelines to improve chronic disease care in low-resource settings. Additionally, the community-based activities provide the added benefit of building a healthy and friendly community environment through community advocacy and participation, and may help to raise awareness and knowledge of chronic diseases within participating villages. Thus, the ComBaCaL project is likely to have a direct positive impact on health outcomes of participants as well as generating evidence to improve context-specific chronic disease care delivery on a longer perspective.

11.3 Participant information and consent

In a first step, before the recruitment of CC-VHWs, the study team will obtain oral village consent from the village chief. Thereafter, participant information and consent seeking will be conducted by the local CC-VHW in a two-stepped approach, first on household and then on individual level.

11.3.1 Household consent

When approaching a household, the CC-VHW will first ask whether the household head or a representative is present. If neither the household head nor a representative is present, the CC-VHW will register the household in the ComBaCaL app together with the name of the absent household head if this one is known.
No further information about any household member will be collected on that day, but the CC-VHW will return to the household within the next days to meet the household head to ask for household consent.
If the household head or a representative is present, the CC-VHW will inform her/him about the content, risks and aims of the study using digital patient information content on the tablet and ask for oral household consent. It will be mentioned specifically, that participation in the ComBaCaL cohort entails consenting to being randomized for future TwiCs and being included in the control population for analysis without further information or being approached for consent to participate in further community-based interventions. To illiterate household heads, texts will be read out or explained, ensuring that the complete information concerning participation is being transmitted. Ample time will be given for consideration and all questions regarding participation will be answered by the CC-VHW.

The household consent will be documented in the ComBaCaL app by the CC-VHW with a checkbox on the electronic household registration form.

11.3.2 Individual consent

If household consent is obtained, the CC-VHW will inform all present household members about the content, risks and aims of the study using the same digital content as for the household head. To illiterate household members, the text will be read out or explained, ensuring that the complete information concerning participation is being transmitted. The ComBaCaL cohort consent is based on the cmRCT approach, i.e. all cohort participants consent to being randomized as part of a future TwiC. In case they are randomized to the control group, they will not be bothered and data collected as part of the cohort study will be used for the TwiC analysis. In case they are randomized to the intervention group, they will be offered the respective intervention, which they may then accept or refuse. Intervention consent will be asked orally, if the intervention is entailing no other than the task-shifting of procedures recommended by the local guidelines to CC-VHWs (i.e. prescription of first-line aHT treatment by CC-VHW). For all other interventions, written informed consent will be sought. Participants may decline any of the offered interventions without implications on further cohort affiliation. Data of participants declining the intervention, but not withdrawing the cohort consent will be included in TwiC analyses. In the participant information, it will be mentioned specifically, that participation in the ComBaCaL cohort includes consenting to being randomized as part of future TwiCs and to being approached for consent to specific interventions. Ample time for consideration will be given to all individuals. The CC-VHW will make sure that all household members will have the opportunity to ask questions about participation in privacy if desired. Individual consent of household members will be documented with an electronic signature of the participant on the study tablet. Illiterate participants will confirm informed consent by drawing a cross in the electronic signature field, countersigned by an impartial witness (may or may not belong to the household).

To children and adolescents under the age of 18 years, the same content will be explained in an age-adapted manner. Written consent will be sought from a guardian aged 18 years or above together with written assent for adolescents aged 10 to 17 years. No written assent will be sought in addition to the guardian’s consent for children below 10 years.

For every household at least one paper-based participant information leaflet will be provided containing all relevant information about study participation as well as contact details of the responsible study staff and representative of the NH-REC to be contacted in case of questions or concerns related to participation.

11.3.3 Withdrawal on household or individual level

Consent can be withdrawn any time on individual or household (by household head) level without justification. Anonymized data collected until the time of withdrawal will be retained in the database.

11.3.4 Service delivery for people declining consent

The CC-VHW will offer the same chronic disease screening and linkage services applied within the study also to inhabitants of the village declining consent to be part of the ComBaCaL main cohort. The data of people not giving consent will not be entered in the ComBaCaL app, but only communicated orally to the individual and noted in the individual’s “Bukana” if desired. By not entering the data in the ComBaCaL app, the clinical decision support for diagnosis will not be available to people declining consent, which might limit the quality of services provided.

11.4 Participant confidentiality

The investigators will ensure that the participants’ confidentiality will be maintained at all times during and after the study, following procedures outlined in section 6.1 (enrolment procedures) and section 8 (data management).

11.5 Participants requiring particular protection

This study has a strong service delivery aspect as the data collected enables case finding and monitoring of the targeted diseases in the study population. Often people with mental or physical conditions impairing capacity for informed consent are particularly vulnerable to the targeted chronic diseases. Therefore, we will offer participation to people with impaired judgement and include them in the ComBaCaL cohort if a guardian provides consent. The incapacity to give informed consent together with the reason will be documented in the electronic ICF together with the guardian's electronic signature (or cross with signature of an impartial witness for illiterate guardians). The same services without the clinical decision support of the ComBaCaL app will be offered to people not capable of giving informed consent for whom no guardian consent is obtained.

11.6 Participant compensation

No compensation will be paid for participation in the study.

12 FUNDING

This research project is funded by the Swiss Agency for Development and Cooperation (SDC) and by the World Diabetes Foundation (WDF), through grants issued to SolidarMed. A written agreement between SolidarMed and Division of Clinical Epidemiology of the University of Basel defines the terms for the collaboration on the research aspects of the project. The funding sources are not involved in the study design, data collection, data analysis, interpretation of the results, or writing the manuscript. The study will be embedded in the SolidarMed Lesotho programme and will thus benefit from logistics and human resources of this organisation. The listed co-investigators have no conflicts of interest.

13 DISSEMINATION OF RESULTS AND PUBLICATION POLICY

13.1 Dissemination to scientific community

International scientific conferences and publications in scientific peer-reviewed journals will serve for wider dissemination of results. Preference will be given to journals with an open-access publication model. Further, anonymised datasets will be made available on open data repositories, such as www.zenondo.org. The study will be registered on ClinicalTrials.gov prior to the start of the trial and a summary of the study protocol will be published in an open-access peer-reviewed journal. The current version of the International Committee of Medical Journal Editors (ICMJE) recommendations is applicable regarding authorship eligibility.[50] The use of professional writers is not intended.

13.2 Information of community and policy makers

Results of this study will be shared with stakeholders at district and national level. In Lesotho, health care workers and stakeholders will be informed about the findings during district meetings headed by the District Health Management Team (DHMT) and at national level, the national research symposium of the MoH and the NCD Technical Working Group will serve as platforms to share the results and discuss their implications among the policy makers.

14 APPENDIX

14.1 Screening and diagnosis of aHT

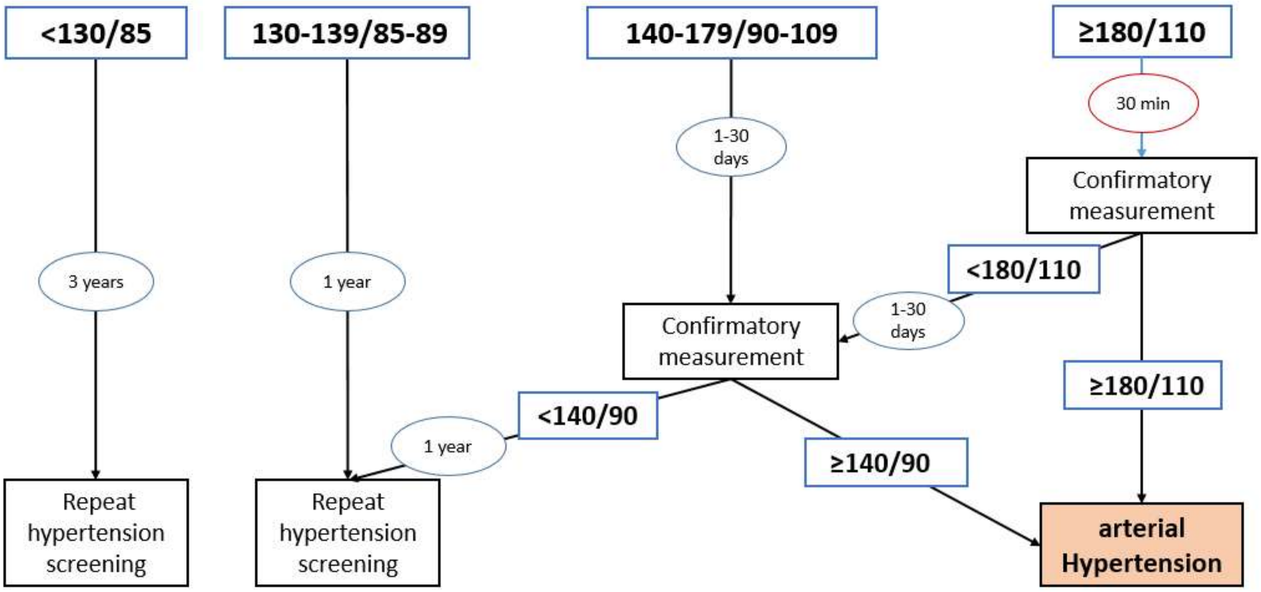

Figure 2 Screening and diagnosis algorithm for aHT

For the determination of BP, standard operating procedure based on the European Society of Cardiology/European Society of Hypertension (ESC/ESH) guidelines 2018[51] and the 2020 Global Hypertension Practice Guidelines of the International Society of Hypertension[52] will be applied using automated BP machines (Omron M3 Comfort [HEM7131-E][53,54]. BP measurements are taken after determination of the correct cuff size in a sitting position after 5 min of rest with feet on the floor, the arm supported without talking or moving during the measurement. At the first visit, the reference arm is determined by measuring BP on both arms. The arm with higher systolic BP is identified as reference arm and used for all subsequent BP measurements. The BP value used for the screening and diagnosis is calculated as the mean value of the last two out of three consecutive measurements at intervals of one minute.
For diagnosis of aHT, two elevated measurements in the range of 140-179/90-109 mmHg on two different days are required or two measurements of 180/110 mmHg or higher on the same day, 30 minutes apart.

14.2 Screening and diagnosis of (pre)DM

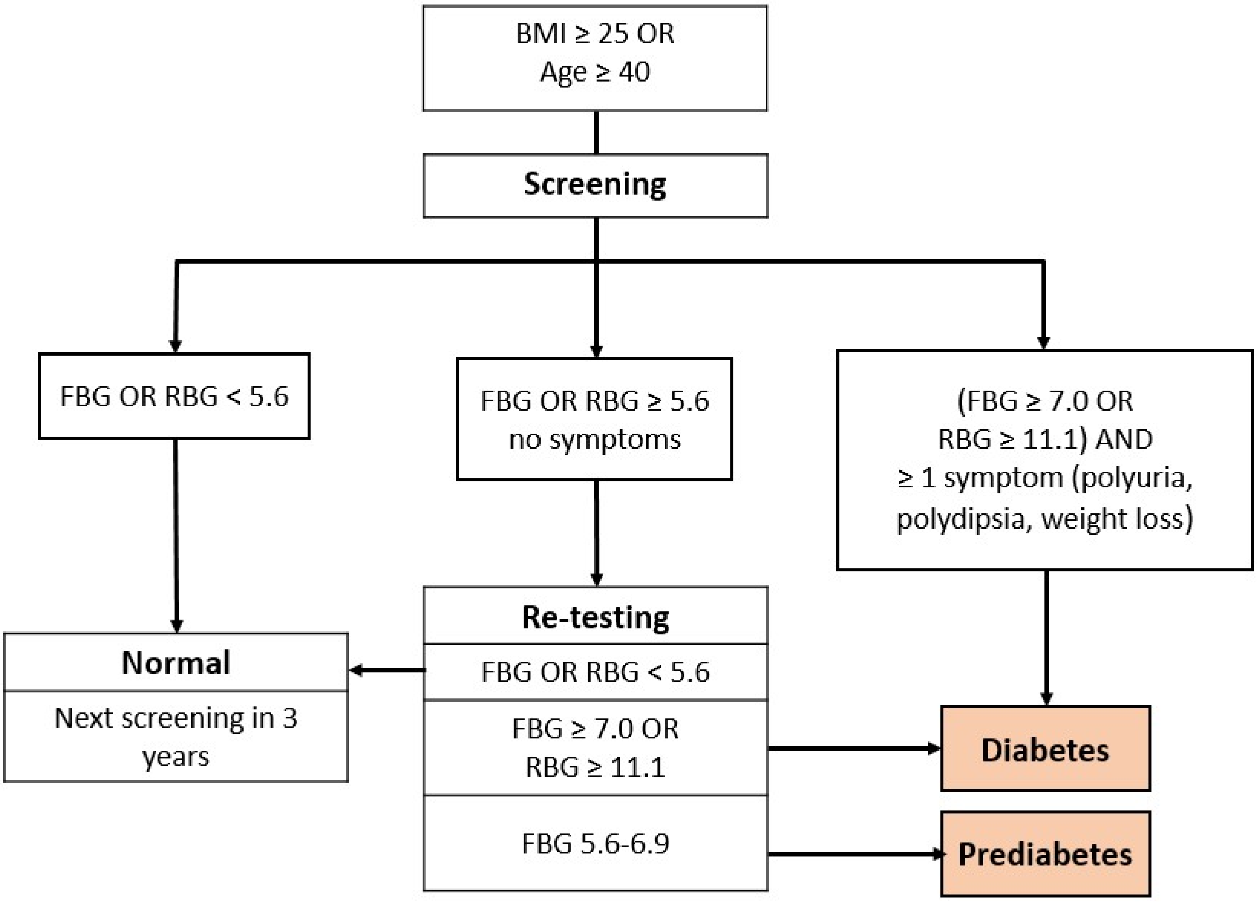

Figure 3 Screening and diagnosis algorithm for (pre)DM. BG: Blood glucose, FBG: Fasting blood glucose, RBG: Random blood glucose.

All adult individuals equal or above 40 years of age or with a BMI equal or above 25kg/m^2 will be screened for (pre)DM using random (RBG) or fasting (FBG) capillary BG measurements. In patients with an FBG or RBG below 5.6 mmol/l, DM can be excluded and the next screening is recommended in three years. Patients with a BG level in the diabetic range (FBG ≥7 mmol/l or RBG ≥11.1 mmol/l) with at least one cardinal symptom of uncontrolled DM (polyuria, polydipsia or weight loss) fulfill the diagnostic criteria for DM. For patients with an elevated BG level, not fulfilling the diagnostic criteria for DM, a confirmatory measurement is required for diagnosis of DM or preDM. The diagnosis of DM is made if the confirmatory measurement is in the diabetic range. PreDM is diagnosed if the FBG is between 5.6 and 6.9 mmol/l.
Patients with an FBG or an RBG value in the diabetic range presenting with all three cardinal symptoms of DM (polyuria, polydipsia or weight loss) will be assessed for ketonuria using urine dip sticks. In case of relevant ketonuria (defined as level 3 or more on a 5 level scale), patients will be immediately referred to a higher-level health facility as they may need admission, insulin treatment and close monitoring.

15 REFERENCES

1 World Health Organization. Noncommunicable diseases country profiles 2018. World Health Organization, 2018 https://apps.who.int/iris/handle/10665/274512 (accessed Sept 13, 2021).

2 Stanaway JD, Afshin A, Gakidou E, *et al.* Global, regional, and national comparative risk assessment of 84 behavioural, environmental and occupational, and metabolic risks or clusters of risks for 195 countries and territories, 1990–2017: a systematic analysis for the Global Burden of Disease Study 2017. *The Lancet* 2018; **392**: 1923–94.

3 Bigna JJ, Noubiap JJ. The rising burden of non-communicable diseases in sub-Saharan Africa. *Lancet Glob Health* 2019; **7**: e1295–6.

4 Gouda HN, Charlson F, Sorsdahl K, *et al.* Burden of non-communicable diseases in sub-Saharan Africa, 1990–2017: results from the Global Burden of Disease Study 2017. *Lancet Glob Health* 2019; **7**: e1375–87.

5 Zhou B, Carrillo-Larco RM, Danaei G, *et al.* Worldwide trends in hypertension prevalence and progress in treatment and control from 1990 to 2019: a pooled analysis of 1201 population-representative studies with 104 million participants. *The Lancet* 2021; **398**: 957–80.

6 Zhou B, Perel P, Mensah GA, Ezzati M. Global epidemiology, health burden and effective interventions for elevated blood pressure and hypertension. *Nat Rev Cardiol* 2021; **18**: 785–802.

7 Noncommunicable Diseases. WHO Reg. Off. Afr. https://www.afro.who.int/health-topics/noncommunicable-diseases (accessed Sept 13, 2021).

8 Joint United Nations Programme on HIV/AIDS. UNAIDS Data 2021. 2021. https://www.unaids.org/en/resources/documents/2021/2021_unaids_data (accessed Dec 14, 2021).

9 Lesotho Millenium Development Goals Status Report 2013. 2013.

10 Labhardt ND, Keiser O, Sello M, *et al.* Outcomes of antiretroviral treatment programmes in rural Lesotho: health centres and hospitals compared. *J Int AIDS Soc* 2013; **16**: 18616.

11 Cohen R, Lynch S, Bygrave H, *et al.* Antiretroviral treatment outcomes from a nurse-driven, community-supported HIV/AIDS treatment programme in rural Lesotho: observational cohort assessment at two years. *J Int AIDS Soc* 2009; **12**: 23.

12 Lewin S, Munabi-Babigumira S, Glenton C, *et al.* Lay health workers in primary and community health care for maternal and child health and the management of infectious diseases. *Cochrane Database Syst Rev* 2010; published online March 17. DOI:10.1002/14651858.CD004015.pub3.

13 Morris MB, Chapula BT, Chi BH, *et al.* Use of task-shifting to rapidly scale-up HIV treatment services: experiences from Lusaka, Zambia. *BMC Health Serv Res* 2009; **9**: 5.

14 Lehmann U, Van Damme W, Barten F, Sanders D. Task shifting: the answer to the human resources crisis in Africa? *Hum Resour Health* 2009; **7**: 49.

15 Joseph JK, Rigodon J, Cancedda C, *et al.* Lay health workers and HIV care in rural Lesotho: a report from the field. *AIDS Patient Care STDS* 2012; **26**: 141–7.

16 Government of Lesotho. Lesotho National Health Strategic Plan 2017-2022. 2016.

17 Firima E, Gonzalez L, Huber J, *et al.* Community-based models of care for management of type 2 diabetes mellitus among non-pregnant adults in sub-Saharan Africa: a scoping review protocol. *F1000Research* 2021; **10**: 535.

18 Fernández LG, Firima E, Robinson E, *et al.* Community-based care models for arterial hypertension management in non-pregnant adults in sub-Saharan Africa: a literature scoping review and framework for designing chronic services. *BMC Public Health* 2022; **22**: 1126.

19 van der Velden JM, Verkooijen HM, Young-Afat DA, *et al.* The cohort multiple randomized controlled trial design: a valid and efficient alternative to pragmatic trials? *Int J Epidemiol* 2016; : dyw050.

20 Declaration of Helsinki https://www.wma.net/policies-post/wma-declaration-of-helsinki-ethical-principles-for-medical-research-involving-human-subjects. https://www.wma.net/policies-post/wma-declaration-of-helsinki-ethical-principles-for-medical-research-involving-human-subjects (accessed May 15, 2022).

21 World Health Organization. Global status report on noncommunicable diseases 2014. World Health Organization, 2014 https://apps.who.int/iris/handle/10665/148114 (accessed Sept 13, 2021).

22 Murray CJL, Aravkin AY, Zheng P, *et al.* Global burden of 87 risk factors in 204 countries and territories, 1990–2019: a systematic analysis for the Global Burden of Disease Study 2019. *The Lancet* 2020; **396**: 1223–49.

23 Mwai GW, Mburu G, Torpey K, Frost P, Ford N, Seeley J. Role and outcomes of community health workers in HIV care in sub-Saharan Africa: a systematic review. *J Int AIDS Soc* 2013; **16**: 18586.

24 Lassi ZS, Bhutta ZA. Community-based intervention packages for reducing maternal and neonatal morbidity and mortality and improving neonatal outcomes. *Cochrane Database Syst Rev* 2015; **2015**. DOI:10.1002/14651858.CD007754.pub3.

25 Bhutta ZA, Lassi ZS, Pariyo G, Huicho L. Global Experience of Community Health Workers for Delivery of Health Related Millennium Development Goals: A Systematic Review, Country Case Studies, and Recommendations for Integration into National Health Systems. .

26 Augustovski F, Chaparro M, Palacios A, *et al.* Cost-Effectiveness of a Comprehensive Approach for Hypertension Control in Low-Income Settings in Argentina: Trial-Based Analysis of the Hypertension Control Program in Argentina. *Value Health* 2018; **21**: 1357–64.

27 World Health Organization. WHO guideline on health policy and system support to optimize community health worker programmes. Geneva: World Health Organization, 2018 https://apps.who.int/iris/handle/10665/352552 (accessed May 20, 2022).

28 UNAIDS and CSD endorse creation of 2 million CHWs in support of 90-90-90 - Center for Sustainable Development. 2017; published online Sept 6. http://csd.columbia.edu/2017/02/17/unaids-and-csd-endorse-creation-of-2-million-chws/ (accessed Sept 6, 2017).

29 Healthcare - Lesotho Review 2015 Edition. 2017; published online June 10. http://www.lesothoreview.com/healthcare-2015.php (accessed June 10, 2017).

30 Kasaie P, Weir B, Schnure M, *et al.* Integrated screening and treatment services for HIV, hypertension and diabetes in Kenya: assessing the epidemiological impact and cost-effectiveness from a national and regional perspective. *J Int AIDS Soc* 2020; **23**. DOI:10.1002/jia2.25499.

31 Sando D, Kintu A, Okello S, *et al.* Cost-effectiveness analysis of integrating screening and treatment of selected non-communicable diseases into HIV/AIDS treatment in Uganda. *J Int AIDS Soc* 2020; **23 Suppl 1**: e25507–e25507.

32 Dave S, Peter T, Fogarty C, Karatzas N, Belinsky N, Pant Pai N. Which community-based HIV initiatives are effective in achieving UNAIDS 90-90-90 targets? A systematic review and meta-analysis of evidence (2007-2018). *PLOS ONE* 2019; **14**: e0219826.

33 Sandholzer-Yilmaz AS, Kroeber ES, Ayele W, Frese T, Kantelhardt EJ, Unverzagt S. Randomised controlled trials on prevention, diagnosis and treatment of diabetes in African countries: a systematic review. *BMJ Open* 2022; **12**: e050021.

34 on behalf of GetReal Work Package 2, Pate A, Candlish J, Sperrin M, Van Staa TP. Cohort Multiple Randomised Controlled Trials (cmRCT) design: efficient but biased? A simulation study to evaluate the feasibility of the Cluster cmRCT design. *BMC Med Res Methodol* 2016; **16**: 109.

35 Relton C, Torgerson D, O'Cathain A, Nicholl J. Rethinking pragmatic randomised controlled trials: introducing the "cohort multiple randomised controlled trial" design. *BMJ* 2010; **340**: c1066.

36 Craig CL, Marshall AL, Sj??Str??M M, *et al.* International Physical Activity Questionnaire: 12-Country Reliability and Validity: *Med Sci Sports Exerc* 2003; **35**: 1381–95.

37 Cultural adaptation - International Physical Activity Questionnaire. https://sites.google.com/site/theipaq/cultural-adaptation (accessed July 11, 2022).

38 Okeyo AP, Seekoe E, de Villiers A, Faber M, Nel JH, Steyn NP. Dietary Practices and Adolescent Obesity in Secondary School Learners at Disadvantaged Schools in South Africa: Urban–Rural and Gender Differences. *Int J Environ Res Public Health* 2020; **17**: 5864.

39 Humeniuk R, Henry-Edwards S, Ali R, Poznyak V, Monteiro MG, Organization WH. The Alcohol, Smoking and Substance involvement Screening Test (ASSIST): manual for use in primary care. 2010; : 68 p.

40 Lu C, Palazuelos D, Luan Y, *et al.* Development assistance for community health workers in 114 low- and middle-income countries, 2007-2017. *Bull World Health Organ* 2020; **98**: 30–9.

41 Humeniuk R, Holmwood C, Beshara M, Kambala A. ASSIST-Y V1.0: First-Stage Development of the WHO Alcohol, Smoking and Substance Involvement Screening Test (ASSIST) and Linked Brief Intervention for Young People. *J Child Adolesc Subst Abuse* 2016; **25**: 384–90.

42 Källmén H, Berman AH, Jayaram-Lindström N, Hammarberg A, Elgán TH. Psychometric Properties of the AUDIT, AUDIT-C, CRAFFT and ASSIST-Y among Swedish Adolescents. *Eur Addict Res* 2019; **25**: 68–77.

43 van der Westhuizen C, Wyatt G, Williams JK, Stein DJ, Sorsdahl K. Validation of the Alcohol, Smoking and Substance Involvement Screening Test in a low- and middle-income country cross-sectional emergency centre study: ASSIST validation in emergency centres. *Drug Alcohol Rev* 2016; **35**: 702–9.

44 Lesotho Bureau of Statistics. 2016 Lesotho population and housing census. 2018.

45 Ministry of Health Lesotho. THE VILLAGE HEALTH PROGRAM POLICY. 2020; : 58.

46 Serina P, Riley I, Stewart A, *et al.* A shortened verbal autopsy instrument for use in routine mortality surveillance systems. *BMC Med* 2015; **13**: 302.

47 Welcome to the Community Health Toolkit Docs Site! Community Health Toolkit. https://docs.communityhealthtoolkit.org/ (accessed May 15, 2022).

48 Wilkinson MD, Dumontier M, Aalbersberg IjJ, *et al.* The FAIR Guiding Principles for scientific data management and stewardship. *Sci Data* 2016; **3**: 160018.

49 Zenodo - Research. Shared. https://zenodo.org/ (accessed Sept 11, 2022).

50 International Committee of Medical Journal Editors. Recommendations for the Conduct, Reporting, Editing and Publication of Scholarly Work in Medical Journals. http://www.ICMJE.org (accessed Dec 19, 2021).

51 Mancia G, Rosei EA, Azizi M, *et al.* 2018 ESC/ESH Guidelines for the management of arterial hypertension. ; : 98.

52 Unger T, Borghi C, Charchar F, *et al.* 2020 International Society of Hypertension Global Hypertension Practice Guidelines. ; : 24.

53 Takahashi H, Yoshika M, Yokoi T. Validation of two automatic devices for the self-measurement of blood pressure according to the ANSI/AAMI/ISO81060-2:2009 guidelines: the Omron BP765 (HEM-7311-ZSA) and the Omron BP760N (HEM-7320-Z). *Vasc Health Risk Manag* 2015; : 49.

54 STRIDE BP. https://stridebp.org/bp-monitors?bb_limitstart=220 (accessed Aug 8, 2022).
