# Supplementary material for: Cohort profile: the open, prospective Community-Based chronic Care Lesotho (ComBaCaL) cohort – design, baseline chronic disease risk factors and hypertension and diabetes care cascades
Source: BMJ Open. 2025 Jul 25;15(7):e093852. doi: 10.1136/bmjopen-2024-093852 (PMC12306284; doi:10.1136/bmjopen-2024-093852)
Supplement: online supplemental file 3 [file bmjopen-15-7-s003.docx]

ComBaCaL – Informed Consent Form – Adults

Today, I would like to invite you to participate in the ComBaCaL cohort study that is going to take place in your village over the next years. ComBaCaL stands for “Community-Based chronic Care Lesotho” and is a project that aims to improve the care for chronic diseases in Lesotho. This Informed Consent Form serves to inform you about the ComBaCaL study, including its purpose, procedures and the risks, benefits and rights associated with participation. In the end of the document, you will be asked whether you would like to participate in the study and to confirm your consent with a signature, if you decide to participate.

The ComBaCaL study is being run by the Ministry of Health of Lesotho, the National University of Lesotho, SolidarMed Lesotho and the University of Basel (Switzerland). It has been approved by the National Health Research Ethics Committee.

### This Informed Consent Form has three parts:

- Part I: Information sheet (to share information about the research with you)
- Part II: Certificate of consent (for signatures if you agree to take part)
- Part III: Statement by the person taking consent

Your household will be given a copy of the information sheet including contacts of responsible study team members.

# PART I: Information Sheet

## Introduction

I am your Chronic Care Village Health Worker (CC-VHW) and I am part of a team conducting research on chronic disease care in Lesotho. I am giving you information about the ComBaCaL study and inviting you to be part of it. There may be some words you do not understand. Please ask me to stop as we go through the information and I will take the time to explain it and answer all the questions you have. If you have questions later, you are free to contact me or the study responsibles whose numbers are mentioned at the end of the form any time.

## Purpose of the ComBaCaL study

The ComBaCaL study seeks to improve chronic disease care in Lesotho with the help of trained CC-VHWs in the community like me. ComBacaL has a special focus on non-communicable diseases (NCDs). These are diseases that cannot be transmitted between people, such as high blood pressure or diabetes. But also other chronic diseases such as HIV and mental health problems are of interest to us. We want to study these diseases and evaluate interventions on how best to prevent, diagnose and treat them in the villages of Lesotho. The CombaCaL study will be implemented in selected villages in Butha-Buthe and Mokhotlong districts.

## Study Procedures

### General Processes

The ComBaCaL study is a cohort study. This means that the participants will be registered in the beginning and then be followed-up over a period of time in the future. Data for this study will be mainly collected by me, the CC-VHW, in the community using an application installed on a tablet.

In a first step, I will register you in the application. After this, data from questionnaires and examinations will be collected and added to your profile in the application. In the future, I will visit you repeatedly for further assessments and updates of previously registered data.

### Type of data collected

The ComBaCaL study is collecting medical information about participants and information about the location, demographics and wealth of participating households.

Participants will be asked questions about their current health condition, their medical history and social determinants, such as their profession or level of education. Standardized questionnaires will be used to assess risk factors for chronic diseases, for example the level of physical activity, the composition of diet or the use of tobacco, alcohol or other substances. Also questions about your mental well-being may be asked.

I will measure the height, weight, blood pressure and abdominal circumference of all adult participants and the blood sugar for those who are 40 years or older or who are overweight. For the blood pressure measurements, I will use an automated machine with an inflatable upper arm cuff. For the measurement of blood sugar, I will do a fingerprick to obtain a few drops of blood for analysis with a glucometer.

I might also ask you to show me your Bukana, so I can register health-related information from there. Also information about your health may be transmitted from the health center nurses to me, which I will then register in the application.

If you are a household head (or representative) you will be asked questions about the composition and wealth status of your household.

Because we want to evaluate the health of participants, I would ask you and your friends or relatives to inform me about serious medical problems that you might encounter during the time of the study, for example, if you need to go to the hospital. I will also record these events in the application.

In the future, additional data might be collected via questionnaires or other examinations. We may ask you to provide blood samples for storage and future analysis. Blood draws via venepuncture will be conducted by trained nurses. You may always refuse to answer specific questions or examinations.

### Follow-up and duration of the study

I will visit all participants at intervals of about half a year to update some of the data collected and to check on the well-being of participants. If participants have medical problems, they may always inform me, so I can visit them earlier. In case, I would be detecting abnormal findings in an assessment, for example an increased blood pressure or high blood sugar, I will offer to come back some days later to confirm the results, so a potential disease could be detected.

The project has not a defined finishing date. If you consent to participation, I (or if I am leaving another CC-VHW) will be visiting you until the project will come to an end or until you decide to stop participation or permanently move out of the village.

### Future interventions

To find out how best chronic diseases should be managed, we will conduct health-related interventions among the participants of the ComBaCaL cohort study. These interventions can entail different activities, such as the delivery of medication in the village or specific counselling. Villages or individuals that are part of the ComBaCaL study will be selected randomly to take part in these interventions, so I cannot tell you now for which interventions you might be selected. Health outcomes of individuals that are being offered a specific intervention will be compared to outcomes of individuals that are not being offered the intervention.

If you get selected for a specific intervention, I will explain you what this intervention is going to be about in a similar way that I am explaining the ComBaCaL study today. You may then freely decide whether you want to accept or refuse the offered intervention. If the offered intervention is consisting of bringing services that are usually available at your health to the community through me, the CC-VHW, I will explain you the risks and benefits of the service offered like the nurse at the health center would and you can then freely decide whether you prefer to receive the services from me or the health center. If you decide to accept the services offered in the community, I will ask you for oral confirmation, but no written confirmation. In case, an intervention consists of services that are not equal to what is being provided by the nurses at the health center, I will ask you to confirm in written that you want to accept the services offered.

If you are not selected for a specific intervention, the regular activities of the ComBaCaL study will continue without any change.

By consenting to participation in the ComBaCaL study, you agree to:

- being visited every 6 to 12 months to ask questions about your health and offer tests for chronic diseases
- being included in the randomization for future interventions
- being offered health related interventions if you get randomly selected for it
- your data being used as a comparison for interventions for which you were not selected.

## Side effects and risks

The assessments and tests used have been approved for the screening of respective conditions and entail minimal risks. We will ask you some sensitive questions about your past medical history as well as other lifestyle questions, which you may feel uncomfortable answering. The examinations such as fingerpricks or blood pressure measurements may cause slight discomfort. If there are specific questions you do not want to answer or if there is an examination you do not want to happen, you are free to refuse. Blood draws via venepuncture may cause discomfort and pose a minimal risk of infection.

## Storage of biological material

We may ask for the collection of blood samples (plasma or dried blood spot) for storage and future analysis. The collection of samples can be refused without impact on the participation in the ComBaCaL cohrt study. Samples collected fall under the biobank agreement (“Biobanking regulations, v2.0”) approved by the ethics committees in Lesotho and Switzerland. Samples will be stored in biobanks in Butha-Buthe or Mokhothlong districts in Lesotho. Samples may only be exported for analysis after submission and approval of a material transport agreement by the National Health Research Ethics Committee. No human genome analysis will be conducted on the samples collected. Results of analysis that are relevant for the health of participants will be communicated to participants. Samples will be destroyed latest five years after termination of the study.

## Benefits and reimbursements

No payment or gifts will be given for participation in this study. Participants will be offered screening and diagnostic services for chronic diseases, such as high blood pressure and diabetes. Early detection and treatment of these diseases may help to prevent future complications. If I would detect a relevant condition during the study activities, I will immediately inform you and recommend you to seek care for it.

Your participation is likely to help us find out how chronic disease can be better managed in the communities of Lesotho and elsewhere.

## Confidentiality

The information you provide is confidential. Your name, address, and other personal information will only be known to persons directly involved in the data collection, such as myself and my direct supervisors, the nurses working at your health center, and very few members of the study team requiring access to it for research purposes. For data verification purposes, the ethics committee may require direct access to parts of your medical data relevant to the study.

Outside of these situations, everything you tell us will remain confidential. For the analysis of the study data, we will only use anonymized data, this means that we will remove all the contact information, so that nobody can find out to which participant the data belongs.

## Sharing of results

We intend to share the knowledge that we get from this research with other researchers around the world and with policy makers in the country. The publication of the results will allow other clinicians, researchers and policy makers to learn from our work. Only results of anonymized data will be shared. This means that neither your name nor any other identifiable information will be accessible to the public.

If you would like to know about the results of the study after the data is analysed, you may contact me or any of the persons listed below.

## Voluntary participation and right to withdraw

Participation in this study is voluntary. It is your choice whether you would like to participate or not. You may refuse to answer any question, examination or sample collection also after you have given consent to participate in the study. You may withdraw your consent to participating in the study at any time. If you refuse to participate or if you withdraw your consent later, this will not have any negative effects on the care you are being offered at the health center. If you wish, you may also receive screening and diagnostic services from me, without being in the study. However, I might not be able to give recommendations of the same quality to people not included in the study, as I am not entering the data into the tablet and thus do not receive guidance by the algorithms coded in the study application. If you decide to participate now and withdraw your consent later, then the data that has been collected until the moment you withdraw from the study will be used for analysis, but no further data will be collected.

## Consent

By participating, you give us permission to register you in the study application and to approach you for the questions, examinations and sample collections mentioned. You also agree to being asked whether you want to receive chronic disease interventions in the future and that your anonymized data may be used to compare it to participants from other villages for specific analysis of interventions taking place in other villages. You also give permission for members of the ethics committee to access your study data collected. Finally, you give us permission to confidentially use the data we collect for this study for additional research in the future.

## Whom to contact

If you have any questions, you may ask them now or later any time during the study. You may always contact me directly or if you wish to speak to the people responsible for the study you may contact the following persons any time: *[table with contact of study staff removed for publication]*

# Part II: Certificate of Consent

#

Participant Name: ____________________________

Statement of consent:

Participant: I have read the previous information or it has been read to me. I have had the opportunity to ask questions and any questions I asked have been answered to my satisfaction. I consent voluntarily to participate in this study

Witnesses: I have witnessed the accurate reading of the consent form to the potential participant or their caregiver, and the individual has had the opportunity to ask questions. I confirm that consent was given freely.

**Signatures:**

| **Participant aged 18 years or older and literate:** | | |
| --- | --- | --- |
| Signature: | | Date (dd.mm.yyyy):  _ _._ _._ _ _ _ |
|  |  |  |
| **Participant aged 18 years or older and illiterate:**    Cross or initials: | | |
| **fWitness:**  *Must be literate and aged 18 years or older. The witness should have no connection to the research team.* | Print Name: | |
|  | Signature: | Date (dd.mm.yyyy):  _ _._ _._ _ _ _ |

# Part III: Statement by the person taking consent

I have accurately read out the information sheet to the potential participant, and/or given them the chance to read it. To the best of my ability, I have made sure that the participant understands the study and its implications. I confirm that the participant was given an opportunity to ask questions about the study, and I have answered all questions correctly and to the best of my ability. I confirm that the individual has not been coerced into giving consent, and that consent has been given freely and voluntarily.

Name: ___________________________________ Date (dd.mm.yyyy): _ _._ _._ _ _ _

Signature: ______________________________
