# Supplementary material for: Cohort profile: the open, prospective Community-Based chronic Care Lesotho (ComBaCaL) cohort – design, baseline chronic disease risk factors and hypertension and diabetes care cascades
Source: BMJ Open. 2025 Jul 25;15(7):e093852. doi: 10.1136/bmjopen-2024-093852 (PMC12306284; doi:10.1136/bmjopen-2024-093852)
Supplement: Supplementary file 5 [file bmjopen-2024-093852.reviewer_comments.pdf]

PEER REVIEW HISTORY

BMJ Open publishes all reviews undertaken for accepted manuscripts. Reviewers are asked to complete a checklist review form and are provided with free text boxes to elaborate on their assessment. These free text comments are reproduced below.

ARTICLE DETAILS

Title (Provisional)

Cohort profile: the open, prospective Community-Based chronic Care Lesotho (ComBaCaL) cohort – design, baseline chronic disease risk factors and hypertension and diabetes care cascades

Authors

Gerber, Felix; Sanchez-Samaniego, Giuliana; Tahirsylaj, Thesar; Lejone, Thabo Ishmael; Lee, Tristan; Raeber, Fabian; Chitja, Mamakhala; Mathulise, Malebona; Kabi, Thuso; Mokaeane, Mosoetsi; Maphenchane, Malehloa; Molulela, Manthabiseng; Mota, Mota; Masike, Sesale; Bane, Matumaole; Makabateng, Retselisitsoe; Khomolishoele, Makhebe; Sematle, Mamoronts'ane; Gupta, Ravi; Ayakaka, Irene; Sao, Lebohang; Tlahali, Mosa; Phaaroe, Sejojo; Litaba, Malitaba; Mphunyane, Madavida; Basler, Dave Brian; Kindler, Kevin; Grimm, Pauline; Seelig, Eleonora; Burkard, Thilo; Briel, Matthias; Chammartin, Frédérique; Amstutz, Alain; Labhardt, Niklaus Daniel D

VERSION 1 - REVIEW

**Reviewer**	**1**
**Name**	**Romero-Sandoval, Natalia**
**Affiliation**	**Universidad Internacional del Ecuador, Quito, School of Medicine**
**Date**	**26-Mar-2025**
**COI**	**None**

Please find the revision detail of the manuscript entitled “Cohort profile: design, sociodemographic characteristics, chronic disease risk factors, and baseline hypertension and diabetes care cascades of the open, prospective Community-Based chronic disease Care Lesotho (ComBaCaL) cohort.”

While this manuscript presents an interesting approach to improving the understanding of NCDs in a low-income country, it requires further refinement. Please find the major observations below for your consideration and that of the authors.

General comment

The proposed work is highly valuable and presents a significant challenge, demonstrating that research can indeed be conducted in difficult settings marked by inequities and marginalisation. This is especially notable in the context of chronic diseases, which pose additional complexities.

However, it is unclear how the role of community health workers was implemented, particularly in their interactions with the citizens. I am keen to understand how this relationship was managed to prevent impositions, colonial dynamics, or power imbalances. In Andean communities of South America, there is a potential for unequal power dynamics between health promoters and the community. I suggest reviewing this article for further insight: Romero-Sandoval, N., Flores-Carrera, O., Molina, M. A., Jácome, M., Navarro, A., & Martin, M. (2009). DOTS strategy and community participation: an experience in the Ecuadorian Andes. *The international journal of tuberculosis and lung disease*, *13*(12), 1569–1571.

Strengths and limitations

Line 71. Community-Based Approach: The term “tailored clinical decision support” is unclear in the context. Throughout this section, the authors should provide more precise and refined information. I suggest clarifying the relationship between “community health workers” and “tailored clinical decision support”, as well as the “approach” and its suggested consequences.

Introduction

Lines 98-99: The word “trials” seems unnecessary, as this approach does not involve the design of epidemiological studies that contribute to prevalences or incidence estimates.

Cohort description

Lines 168-172: I suggest that the authors specify how they would apply informed consent if selected for future nested trial(s), either as a participant or as a control. I suggest revising the paper:

Amstutz, A., Schönenberger, C. M., Speich, B., Griessbach, A., Schwenke, J. M., Glasstetter, J., James, S., Verkooijen, H. M., Nickolls, B., Relton, C., Hemkens, L. G., Chammartin, F., Gerber, F., Labhardt, N. D., Schandelmaier, S., & Briel, M. (2024). Characteristics, consent patterns, and challenges of randomized trials using the Trials within Cohorts (TwiCs) design - A scoping review. *Journal of Clinical Epidemiology*, *174*, 111469. https://doi.org/10.1016/j.jclinepi.2024.111469

Lines 196-205. Which is the native language in participant communities? Is feasible to quantity the proportion of inhabitants who speak / understand English? This information could enhance the clarity and coherence of the study setting, although a statement is already present in line 299.

Lines 280-281. Although the authors include the IPAQ-SF questionnaire as an instrument for collecting information on physical activity, it is recommended that the conceptual framework addressing potential biases in its implementation be explicitly stated. Ideally, this should incorporate local or regional data to provide context for the reader. Additionally, I suggest that the research team specify the characteristics of the group administering the questionnaire, particularly given its application in predominantly rural populations with high poverty rates. Alternatively, if any adaptations will be made to align with the population's worldview, this should be clarified. Similar considerations apply to dietary questionnaires.

Line 296. How was collected the “current medication intake”? Some studies about households aid kit recommend applying the direct observation of first-aid kit used at home.

Line 305-306. Specify the threshold for "higher" blood pressure by defining the cut-off value to identify as reference arm.

Line 318. Specify the fasting period.

Findings to date

Lines 368-383 The paragraph is quite lengthy, as it largely reiterates the information already presented in table 1. Consider summarising key points more concisely to improve readability.

References

I would like to suggest that the authors review the references because they could be reduced in order to facilitate reading in a comprehensive and more rational way.

Reviewer 2

Name Beidelman, Erika T.

Affiliation Indiana University Bloomington School of Public Health, Department of Epidemiology and Biostatistics

Date 06-Apr-2025

COI None

The authors present the cohort profile and description of the study population for a chronic disease cohort platform in Lesotho. This cohort is incredibly robust with nearly 100% population coverage. It is also novel in its employment of trained CHWs for data collection and ability to evaluate integrated trial experiments. The authors provide comprehensive detail on the platform but need to make a few points regarding their study design clearer. This includes their intervals for data collection as well as information surrounding the final

count of included villages. Overall, the authors may want to consider streamlining the presentation and discussion of results to better focus on chronic disease risk factors and the hypertension/diabetes care cascades as these are described as their main priorities.

- I would recommend re-working your title to something a bit more streamlined.
- Lines 118-126: It would be helpful if you specifically state the gaps that the cohort aims to fill and its goals. You give a brief description of collected information but what is the goal of having that information outside of the evaluation of chronic care models that you mention? Is the only aim to assess chronic care models?
- Lines 144-159: Some of this info seems like it would be good to put in your Introduction to describe the need for the ComBaCaL cohort in Lesotho. High HIV and NCD burden, but NCD care is not integrated into the CHW system � ComBaCaL will allow for assessment of novel delivery models?
- Line 160 - Objectives and Design: This section is called Design but does not include information about the mode of data collection (household surveys, individual questionnaires, etc…?) or the rate of data collection/how often follow-up will occur. Your title mentions this is an open cohort but that information is missing here. Is data only collected when there is an ongoing trial or at regular intervals? This isn't clear here. Some of this information is provided in detail in later sections, but a high-level overview should be described in the Design section.
- Line 175: This is the first time that it is made clear that CHWs are sampled for participation rather than households or residents. Please add something to the Objectives and Design section that describes the various units of observation under the cohort (e.g., households, individual residents, CHWs, etc…) and whether CHWs are collecting your data.
- Lines 194-195: Directly state your final sample of villages not just the number excluded.
- Line 218: I am understanding from this section that the cohort is aiming for total population coverage of residents from sampled villages. If so, this is a big strength of the cohort and I would be sure to mention it in the Introduction and in the Objectives and Design section. Additionally, can you present total enrollment counts and % of population participating in this section?
- Line 228: What information is recorded regarding households/household members who do not provide informed consent? Do you document the number of households/household members?
- Lines 245-252: At what interval are CHWs visiting households? Annual visits? How are CHWs made aware of deaths/emigration?
- Lines 255-259: Did all CHWs fully attend all trainings?
- Line 254 – Measurements: Can you divide this into sub-sections to describe the types of collected data? There is a lot of information presented and it gets a bit confusing.

• Line 339: How did you arrive at 103 from the sampled 140 villages? In the Methods I only see that 29 were excluded and 111 had CHWs participating in trainings.

• You collected abdominal circumference but mainly report on BMI instead of waist-to-hip ratio. Waist-to-hip ratio is often considered to be a better indicator of overall and cardiovascular health risk. Could you report on this as well?

• Strengths and Limitations – You fail to mention your 100% population coverage and collection of mortality and migration data as a major strength. This majorly reduces the risk of bias from self-selection on your prevalence estimates. It also gives you the means to adjust for the effects of differential attrition and mortality in future longitudinal studies. Additionally, can you speak to any limitations caused by using local CHWs, like social desirability bias?

• Is Figure 1 included in your main paper or supplementary materials? I didn't see it referenced in the main paper, but it would be helpful to include it there. The figure is also missing the year in the second to last box which states "103 villages with trained CHW at study start in".

VERSION 1 - AUTHOR RESPONSE

Reviewer 1 Comments:

- General comment: The proposed work is highly valuable and presents a significant challenge, demonstrating that research can indeed be conducted in difficult settings marked by inequities and marginalisation. This is especially notable in the context of chronic diseases, which pose additional complexities. However, it is unclear how the role of community health workers was implemented, particularly in their interactions with the citizens. I am keen to understand how this relationship was managed to prevent impositions, colonial dynamics, or power imbalances. In Andean communities of South America, there is a potential for unequal power dynamics between health promoters and the community. I suggest reviewing this article for further insight: Romero-Sandoval, N., Flores-Carrera, O., Molina, M. A., Jácome, M., Navarro, A., & Martin, M. (2009). DOTS strategy and community participation: an experience in the Ecuadorian Andes. The international journal of tuberculosis and lung disease, 13(12), 1569–1571.
    - Thank you very much for the appreciative comments and highlighting important insights from the work conducted in South America. Personal relationships are fundamental in health service delivery, and issues, such as impositions and power imbalances should be carefully addressed.
    - In Lesotho, Community Health Workers (CHWs) have been a trusted cadre since 1978. The ComBaCaL cohort is fully integrated within the existing CHW system. Recruitment of new CHWs in villages were there was no pre-existing CHW was conducted according to the national policies through an election by the community members organized by the Ministry of Health.
    - We recognize that factors influencing personal relationships and power dynamics, such as the level of responsibility assigned to CHWs, their involvement in research tasks like consent-seeking, and the implementation of digital clinical decision support, may differ from routine settings. To explore this further, we have investigated the influence of digital clinical decision support on client interactions from the CHW perspective in a separate publication (1).

Additionally, we are currently conducting a mixed-methods assessment among participants with hypertension and/or diabetes to explore their perceptions on CHW-led services for these conditions.

- The main goal of the cohort profile manuscript is to describe the design, implementation, and baseline characteristics of the cohort study. The relevant information regarding CHW recruitment and training, as well as participant enrolment and data collection are provided in the manuscript. Given that the manuscript is quite extensive already and considering that assessments of CHWclient interactions are described in separate manuscripts, we consider these aspects out of scope for this manuscript.

- Strengths and limitations: Line 71. Community-Based Approach: The term "tailored clinical decision support" is unclear in the context. Throughout this section, the authors should provide more precise and refined information. I suggest clarifying the relationship between "community health workers" and "tailored clinical decision support", as well as the "approach" and its suggested consequences.
  - The statement in the "Strengths and limitations" section has been adapted to enhance clarity: "Data is captured by local Community Health Workers (CHWs) residing in the study villages using a tablet-based clinical decision support and data collection application. Through repeated home visits by CHWs, and remote monitoring by study staff, nearly all inhabitants of the study villages were enrolled and high levels of data quality and completeness were achieved."

- Introduction Lines 98-99: The word "trials" seems unnecessary, as this approach does not involve the design of epidemiological studies that contribute to prevalences or incidence estimates.
  - The sentence has been revised and the word "trials" removed.

- Cohort description: Lines 168-172: I suggest that the authors specify how they would apply informed consent if selected for future nested trial(s), either as a participant or as a control. I suggest revising the paper: Amstutz, A., Schönenberger, C. M., Speich, B., Griessbach, A., Schwenke, J. M., Glasstetter, J., James, S., Verkooijen, H. M., Nickolls, B., Relton, C., Hemkens, L. G., Chammartin, F., Gerber, F., Labhardt, N. D., Schandelmaier, S., & Briel, M. (2024). Characteristics, consent patterns, and challenges of randomized trials using the Trials within Cohorts (TwiCs) design - A scoping review. Journal of Clinical Epidemiology, 174, 111469.
  https://doi.org/10.1016/j.jclinepi.2024.111469 o Several investigators of the ComBaCaL cohort study are co-authors of the mentioned manuscript. A sentence has been added to clarify the procedures for the intervention consent: "Intervention consent will be asked orally, if the intervention is entailing no other than the task-shifting of procedures recommended by the local guidelines. For all other interventions, written intervention consent will be required."

- Lines 196-205. Which is the native language in participant communities? Is feasible to quantity the proportion of inhabitants who speak / understand English? This information could enhance the clarity and coherence of the study setting, although a statement is already present in line 299.
  - The two official languages in Lesotho are Sesotho and English. For most people, Sesotho is the primary language, but all schools are teaching in English from the first year of primary school. The entire content of our application was provided in English and Sesotho. Community Health Workers usually talk Sesotho to participants. The following clarification has been added: "The official languages of Lesotho are English and Sesotho. Sesotho is the primary language spoken by most of the population, while all subjects in schools are typically taught in English from the first year of primary education."

- Lines 280-281. Although the authors include the IPAQ-SF questionnaire as an instrument for collecting information on physical activity, it is recommended that the conceptual framework addressing potential biases in its implementation be explicitly stated. Ideally, this should incorporate local or regional data to provide context for the reader. Additionally, I suggest that the research team specify the characteristics of the group administering the questionnaire, particularly given its application in predominantly rural populations with high poverty rates. Alternatively, if any adaptations will be made to align with the population's worldview, this should be clarified. Similar considerations apply to dietary questionnaires.
    - Thank you for this valid comment. The IPAQ-SF questionnaire has been adapted to the local context according to official recommendations (https://sites.google.com/view/ipaq/adapt). To ensure conceptual, metric and linguistic equivalence, the questions were discussed with the local study team and community members. A version using culturally adapted terms in English was developed and refined through translation and back-translation. The developed questionnaire was pilottested and then used in a large-scale population-based survey in the same region (2). The study nurses and research assistants involved in the survey were responsible for the training of the CHWs involved in the ComBaCaL cohort study. All questions were provided in English and Sesotho. The following sentence has been added to the “Measurements” section: “The IPAQ-SF has been adapted to the local context according the official instructions, including translation and backtranslation (3).”
    - The food frequency questionnaire is based on and an assessment tool for obesity that has been used previously in South Africa (4). To ensure local contextualization, the food items were selected to represent the most frequently consumed unhealthy food items in the study region. This is explained in the “Measurements” section.

- Line 296. How was collected the “current medication intake”? Some studies about households aid kit recommend applying the direct observation of first-aid kit used at home.
    - The following sentence in the “Measurements” section describes the collection of clinical information including medication use: “For adults aged 18 years and above, (…) medication use is documented. For the documentation of clinical information, including medication use, self-reports and the participants’ personal health booklets were used”. To clarify that we did not collect data on the direct intake of medication but on the self-reported current medication use, the word 'intake' has been replaced by 'use' when referring to data on medication. Similarly, the word ‘intake’ has been replaced by the word 'consumption' when referring to data on food or beverages throughout the manuscript.

- Line 305-306. Specify the threshold for "higher" blood pressure by defining the cut-off value to identify as reference arm.
    - “Higher” refers to the comparison between the two arms, independent of the absolute value. To enhance clarity, we have adapted the punctuation and wording of the sentence in the “Measurements” section as follows: “At the first visit, the reference arm is determined by measuring blood pressure on both arms; the arm with the higher systolic blood pressure is identified as reference arm and used for all subsequent measurements.”

- Line 318. Specify the fasting period.
    - The following sentence was added in the “Measurements” section: “Fasting was defined as no caloric intake within the preceding eight hours.”

- Findings to date: Lines 368-383 The paragraph is quite lengthy, as it largely reiterates the information already presented in table 1. Consider summarising key points more concisely to improve readability.

- o The paragraph has been shortened to focus on summarizing key points more concisely: “Table 1 provides an overview of socioeconomic indicators at household level, the complete household-level data is available in the supplementary file S2. The median asset-based IWI score is 26 (IQR 17–36), and the monetary monthly household income is $42.4 USD (IQR $18.6–63.6). The main source of drinking water is a community tap (81.4%). Toilet facilities mostly consist of pit latrines (47.6%) while 39.1% do not have a toilet and defecate in fields or bushes. Only 13.2% of households have access to electricity. Cooking and heating primarily rely on wood (84.1% and 63.9%) and animal dung (6.0% and 26.4%). Roughly half of households (50.9%) have geographically difficult access to a health facility, defined as living more than ten kilometers away from the closest health facility or having to cross a river or a mountain to reach the facility. 22.4% of households report inability to access healthcare services despite needing it, mainly due to cost or transport issues. 62.4% of households own agricultural land and for 24.2% of households, farming is the main source of income.”

- References: I would like to suggest that the authors review the references because they could be reduced in order to facilitate reading in a comprehensive and more rational way.
  - o All references have been reviewed, and non-essential ones have been removed.

Reviewer 2 Comments:

- Comments to the Author: The authors present the cohort profile and description of the study population for a chronic disease cohort platform in Lesotho. This cohort is incredibly robust with nearly 100% population coverage. It is also novel in its employment of trained CHWs for data collection and ability to evaluate integrated trial experiments.
  - o Thank you very much for the appreciative feedback.

- The authors provide comprehensive detail on the platform but need to make a few points regarding their study design clearer. This includes their intervals for data collection as well as information surrounding the final count of included villages.
  - o The following sentence has been added in the “Sampling of villages and recruitment of CHWs” section to enhance clarity regarding the final count of included villages: “Of the 111 CHWs invited, 103 passed the training (1 did not attend, 3 did not pass, and 4 chose not to participate), resulting in a final sample of 103 villages included in the study.”

- Overall, the authors may want to consider streamlining the presentation and discussion of results to better focus on chronic disease risk factors and the hypertension/diabetes care cascades as these are described as their main priorities.
  - o This manuscript aims to describe the setting, design, implementation, and baseline characteristics of the Community-Based chronic Care Lesotho cohort. The hypertension and diabetes care cascades are important baseline findings that will be discussed in more detail in separate publications. One manuscript assessing the coverage and costs of CHW-led hypertension and diabetes screening and another one investigating the hypertension care cascade in more details including the analysis of factors associated with untreated, uncontrolled, and overtreated hypertension are currently under review at other journals (5). Furthermore, the nested trials on hypertension and diabetes care have recently been successfully completed, will be published soon and present in-depth data on these two chronic diseases (6,7). This cohort profile is the only manuscript that allows for a more detailed description of broader socioeconomic and behavioral health determinants of the setting and our cohort population. Therefore, we suggest keeping the scope of this manuscript as it is.

- I would recommend re-working your title to something a bit more streamlined.
  - The title was changed to: “Cohort profile: the open, prospective Community-Based chronic Care Lesotho (ComBaCaL) cohort – design, baseline chronic disease risk factors and hypertension and diabetes care cascades”

- Lines 118-126: It would be helpful if you specifically state the gaps that the cohort aims to fill and its goals. You give a brief description of collected information but what is the goal of having that information outside of the evaluation of chronic care models that you mention? Is the only aim to assess chronic care models?
  - To clarify the aims and value of the ComBaCaL cohort study, the following sentence has been added to the “Introduction” section: “Addressing the evidence gaps on the burden and risk factors of chronic diseases in remote rural areas, the Community-Based Chronic Care Lesotho (ComBaCaL) cohort study provides population-based data on the prevalence of chronic diseases and their socioeconomic and behavioral determinants in rural Lesotho. These data are essential for guiding the development of tailored community-based strategies to address the growing chronic disease burden and to optimize health system resource allocation.”

- Lines 144-159: Some of this info seems like it would be good to put in your Introduction to describe the need for the ComBaCaL cohort in Lesotho. High HIV and NCD burden, but NCD care is not integrated into the CHW system ComBaCaL will allow for assessment of novel delivery models?
  - Due to the scarcity of population-level data on chronic diseases and their risk factors in rural areas across most low- and middle-income countries, the ComBaCaL cohort data is of relevance beyond Lesotho. Understanding the local setting is essential for the interpretation of the findings. We have decided to focus the introduction on the broader background on chronic disease burden, risk factors and evidence gaps across low- and middle-income countries, while introducing the local context in a specific paragraph within the “Cohort description” section. After careful consideration and discussion among the study team, we do not think, it will improve the readability of the manuscript to merge the local setting with the general introduction but suggest to keep it in a separate paragraph at the beginning of the ”Cohort description” section.

- Line 160 - Objectives and Design: This section is called Design but does not include information about the mode of data collection (household surveys, individual questionnaires, etc…?) or the rate of data collection/how often follow-up will occur. Your title mentions this is an open cohort but that information is missing here. Is data only collected when there is an ongoing trial or at regular intervals? This isn’t clear here. Some of this information is provided in detail in later sections, but a high-level overview should be described in the Design section.
  - The following sentences have been added to the “Objectives and Design” section:
    - “The ComBaCaL cohort is an open, population-based, prospective research and service delivery platform, with the aim to assess the prevalence, burden, and risk factors of chronic diseases, especially arterial hypertension, type 2 diabetes, and HIV in rural Lesotho. It aims to enroll all consenting individuals of randomly selected rural villages and to assess chronic disease burden and risk factors over time. Data is collected by trained CHWs during home visits. There is a continuous census update with enrolment of people being born or moving into the villages and censoring of participants who die or move out of the village.”
    - “Follow-up data collection of the cohort is flexible and influenced by the nested trials. Besides the continuous census updates and the follow-up of participants with conditions of interest as part of nested trials, there will be two-yearly population-based follow-up visits.”

- Line 175: This is the first time that it is made clear that CHWs are sampled for participation rather than households or residents. Please add something to the Objectives and Design section that describes the various units of observation under the cohort (e.g., households, individual residents, CHWs, etc…) and whether CHWs are collecting your data.
    - This has been addressed in the statement above.

- Lines 194-195: Directly state your final sample of villages not just the number excluded.
    - The following sentence has been added in the section "Sampling of villages and recruitment of CHWs": "Of the 111 CHWs invited, 103 passed the training (1 did not attend, 3 did not pass, and 4 chose not to participate), resulting in a final sample of 103 villages included in the study."

- Line 218: I am understanding from this section that the cohort is aiming for total population coverage of residents from sampled villages. If so, this is a big strength of the cohort and I would be sure to mention it in the Introduction and in the Objectives and Design section. Additionally, can you present total enrollment counts and % of population participating in this section?
    - The following statement has been added to the "Objectives and Design section": "The ComBaCaL cohort is an open, population-based, prospective research and service delivery platform, with the aim to assess the prevalence, burden, and risk factors of chronic diseases, especially arterial hypertension, type 2 diabetes, and HIV in rural Lesotho. It aims to enroll all consenting individuals of randomly selected rural villages and to assess chronic disease burden and risk factors over time."
    - In the "Participants' characteristics" section, the following sentence was adapted to present total enrollment counts and percentages of population participating: "Since February 2023, in the 103 study villages, 5'274 households with 16'461 people were approached for household consent to participate in the ComBaCaL cohort. Through repeated home visits by the CHWs and verification by the study team, nearly all households of the 103 villages were reached. For 11 households (0.2%) with 31 people, household consent was refused. 16'430 individuals from 5'263 households were approached for individual consent. 16'158 participants (98.2% of overall 16'461 eligible) consented to participation in the cohort study."

- Line 228: What information is recorded regarding households/household members who do not provide informed consent? Do you document the number of households/household members?
    - Yes, the number of households and household members who do not give consent is collected and reported in the section "Participants' characteristics"

- Lines 245-252: At what interval are CHWs visiting households? Annual visits? How are CHWs made aware of deaths/emigration?
    - The following statement has been added to the "Objectives and Design" section: "Follow-up data collection of the cohort is flexible and influenced by the nested trials. Besides the continuous census updates and the follow-up of participants with conditions of interest as part of nested trials, there will be two-yearly population-based follow-up visits."
    - The villages have an average size of roughly 50 households, hence the CHWs know all inhabitants and have overview of people dying or migrating.
    - The following paragraph has been added to the "Measurements" section: "CHWs continuously document census updates (births, deaths, migration, withdrawals) and relevant clinical events any time during the cohort follow-up. Clinical events documented include serious clinical events (deaths, hospitalizations, events leading to disability or congenital anomaly) and clinical events of special interest (stroke, myocardial infarction, heart failure,

chronic kidney disease, diabetic foot syndrome, peripheral arterial disease, hypertensive urgency or emergency, hyperglycemic emergency, vision loss, peripheral neuropathy). CHW may solicit clinical events, births, and migrations through reporting by participants, friends or relatives, screening of participants' personal health booklets (Bukanas) or reporting by staff from health care facilities or other VHWs. Clinical event reports submitted by CHWs via the ComBaCaL app are verified remotely by the supervising study nurxe. The anonymized reports are subsequently submitted to a blinded study physician for classification as serious clinical event, clinical event of special interest or neither of the two.

The content and timing of follow-up data collection is influenced by the nested trials. Participants enrolled in nested trials will receive follow-up visits for delivery of trial interventions and data collection according to the corresponding protocols [31,32]. Hypertension and diabetes screening follow-ups are scheduled every three years for participants with a negative screening at baseline. Participants with a high-normal BP (130-139/85-89 mmHg), are followed-up after six months. Participants with a BG in the prediabetic range (FBG 5.6-6.9 mmol/l after previously elevated BG) are followed-up after two months. A population-level follow-up for the update of sociodemographic and health data is scheduled every two years."

- Lines 255-259: Did all CHWs fully attend all trainings?
  - Yes. Few CHWs have missed parts of the official training due to pregnancy or sickness and were then trained individually to ensure that they have been trained on the full content.

- Line 254 – Measurements: Can you divide this into sub-sections to describe the types of collected data? There is a lot of information presented and it gets a bit confusing.
  - The section has been divided into sub-sections and several sentences have been reworded to enhance clarity.

- Line 339: How did you arrive at 103 from the sampled 140 villages? In the Methods I only see that 29 were excluded and 111 had CHWs participating in trainings.
  - The following sentence has been added in the "Sampling of villages and recruitment of CHWs" section: "Of the 111 CHWs invited, 103 passed the training (1 did not attend, 3 did not pass, and 4 chose not to participate), resulting in a final sample of 103 villages included in the study."

- You collected abdominal circumference but mainly report on BMI instead of waist-to-hip ratio. Waist-tohip ratio is often considered to be a better indicator of overall and cardiovascular health risk. Could you report on this as well?
  - Unfortunately, we only collected abdominal circumference without hip circumference. Therefore, we can only report on abdominal circumference.

- Strengths and Limitations – You fail to mention your 100% population coverage and collection of mortality and migration data as a major strength. This majorly reduces the risk of bias from self-selection on your prevalence estimates. It also gives you the means to adjust for the effects of differential attrition and mortality in future longitudinal studies. Additionally, can you speak to any limitations caused by using local CHWs, like social desirability bias?
  - The following statements have been added to the "Strengths and limitations" section: "Through enrolment and data collection conducted by CHWs residing in the study villages, nearly all inhabitants are included, and exceptionally high levels of data completeness and screening coverage are achieved. Continuous census updates with documentation of deaths, births and

migration ensure that the ComBaCaL cohort remains representative of the village populations at all time points."

Is Figure 1 included in your main paper or supplementary materials? I didn't see it referenced in the main paper, but it would be helpful to include it there. The figure is also missing the year in the second to last box which states "103 villages with trained CHW at study start in". o Thank you for spotting this mistake. The figure is now corrected and referenced in the manuscript, in the section "Sampling of villages and recruitment of CHWs".

Furthermore, we have corrected two minor mistakes in table 1 and supplementary table 2. In table 1, we made a mistake in the calculation of the food insecurity categories. After correction, the prevalence of food insecurity is slightly higher than reported previously. In the supplementary table 2, we corrected the age categories to only show the age distribution among adult participants as the other outcomes reported in that table only refer to adult par-
ticipants.

References

1. Staehelin D, Schmid D, Gerber F, Dolata M, Schwabe G. Empowering Community Health Workers with Scripted Medicine: A Design Science Research Study (Preprint) [Internet]. 2024 [cited 9. April 2024]. available at:
http://preprints.jmir.org/preprint/57545

2. Fernández LG, Firima E, Gupta R, Sematle MP, Khomolishoele M, Molulela M, u. a. Prevalence and determinants of cardiovascular risk factors in Lesotho: a population-based survey. Int Health. August 2023;

3. Geisinger KF. Cross-cultural normative assessment: Translation and adaptation issues influencing the normative interpretation of assessment instruments. Psychol Assess. 1994;6(4):304–12.

4. Okeyo AP, Seekoe E, de Villiers A, Faber M, Nel JH, Steyn NP. Dietary Practices and Adolescent Obesity in Secondary School Learners at Disadvantaged Schools in South Africa: Urban–Rural and Gender Differences. Int J Environ Res Public Health. 13. August 2020;17(16):5864.

5. Spaolonzi N, Gupta R, Sanchez-Samaniego G, Carter A, Tahirsylaj T, Raeber F, u. a. Costs and Coverage of Community Health Worker-Led Hypertension and Diabetes Screening in Rural Lesotho: A Cohort Study with a Cost-Consequence Analysis [Internet]. Rochester, NY: Social Science Research Network; 2025 [cited 13. April 2025]. available at: https://papers.ssrn.com/abstract=5177608

6. Gerber F, Gupta R, Lejone TI, Tahirsylaj T, Lee T, Sanchez-Samaniego G, u. a. Community-based management of arterial hypertension and cardiovascular risk factors by lay village health workers for people with controlled and uncontrolled blood pressure in rural Lesotho: joint protocol for two cluster-randomized trials within the ComBaCaL cohort study (ComBaCaL aHT Twic 1 and ComBaCaL aHT TwiC 2). Trials. 6. Juni 2024;25(1):365.

7. Gerber F, Gupta R, Lejone TI, Tahirsylaj T, Lee T, Kohler M, u. a. Community-based type 2 diabetes care by lay village health workers in rural Lesotho: protocol for a cluster-randomized trial within the ComBaCaL cohort study (ComBaCaL T2D TwiC). Trials. 24. Oktober 2023;24(1):688.

VERSION 2 - REVIEW

**Reviewer**	**1**
**Name**	**Romero-Sandoval, Natalia**
**Affiliation**	**Universidad Internacional del Ecuador, Quito, School of Medicine**
**Date**	**11-Jun-2025**
**COI**	

The authors have considered and responded all comments.
